# EccDNAs are apoptotic products with high innate immunostimulatory activity

**DOI:** 10.1038/s41586-021-04009-w

**Published:** 2021-10-20

**Authors:** Yuangao Wang, Meng Wang, Mohamed Nadhir Djekidel, Huan Chen, Di Liu, Fred Alt, Yi Zhang

**Affiliations:** 1 Howard Hughes Medical Institute, 200 Longwood Av., Boston, MA 02115.; 2 Program in Cellular and Molecular Medicine, Boston Children’s Hospital, 200 Longwood Av., Boston, MA 02115.; 3 Department of Genetics, 200 Longwood Av., Boston, MA 02115.; 4 Wyss Institute for Biologically Inspired Engineering, Harvard University, 200 Longwood Av., Boston, MA 02115.; 5 Department of Systems Biology, Harvard Medical School, 200 Longwood Av., Boston, MA 02115.; 6 Harvard Stem Cell Institute WAB-149G, 200 Longwood Av., Boston, MA 02115.

## Abstract

Extrachromosomal circular DNA elements (EccDNAs) have been described in the literature for several decades, and are known for their broad existence across different species^1,2^. However, their biogenesis, and functions are largely unknown. By developing a new circular DNA enrichment method, here we purified, and sequenced full-length eccDNAs with Nanopore sequencing. We found that eccDNAs are mapped across the entire genome in a close to random fashion, suggesting a biogenesis mechanism of random ligation of genomic DNA fragments. Consistently, we found that apoptosis inducers can increase eccDNA generation, which is dependent on apoptotic DNA fragmentation followed by ligation by the DNA ligase 3. Importantly, we demonstrated that eccDNAs can function as potent innate immunostimulants in a sequence-independent, but circularity, and cytosolic DNA sensing Sting-dependent fashion. Collectively, our study not only revealed the origin, biogenesis, and immunostimulant function of eccDNAs, but also uncovered their sensing pathway and potential clinical implications in immune response.

Since its first description in wheat embryos and boar sperm in 1964^3^, extrachromosomal circular DNA (eccDNA) has been reported in almost all cell lines and tissues^4^ across different species although their abundance is highly variable^1,2^. Unlike the independently existing circular DNA in organelles, such as mitochondrial DNA (mtDNA), eccDNAs are derived from genomic DNA with sizes ranging from a few hundred bases to mega bases^1^. Although some studies have suggested that eccDNA generation might be linked to DNA damage repair^5^, hyper-transcription^5,6^, homologous recombination^7^, and replication stress^5^, how exactly eccDNAs are generated is largely unknown. Similarly, it is also unclear whether eccDNA has any function, although some studies have suggested that eccDNAs might contribute to gene amplification in cancer^1^, or might be linked to aging^6,7,8^.

To understand the eccDNA biogenesis, efficient and robust methods that allow purification and sequencing of eccDNAs are needed. Most existing eccDNA purification procedures involve two sequential steps, isolation of crude extrachromosomal DNA followed by removing contaminating linear DNA through exonuclease digestion^5,6,9^, and rolling cycle amplification (RCA) for profiling^5,9,10^. However, most eccDNA samples prepared this way contain a high level of linear DNA before RCA as revealed by electron microscope^5,9^, indicating exonuclease digestion alone is not sufficient to eliminate all contaminating linear DNA.

## An efficient eccDNA purification method

**Extended Data Fig. 1.**
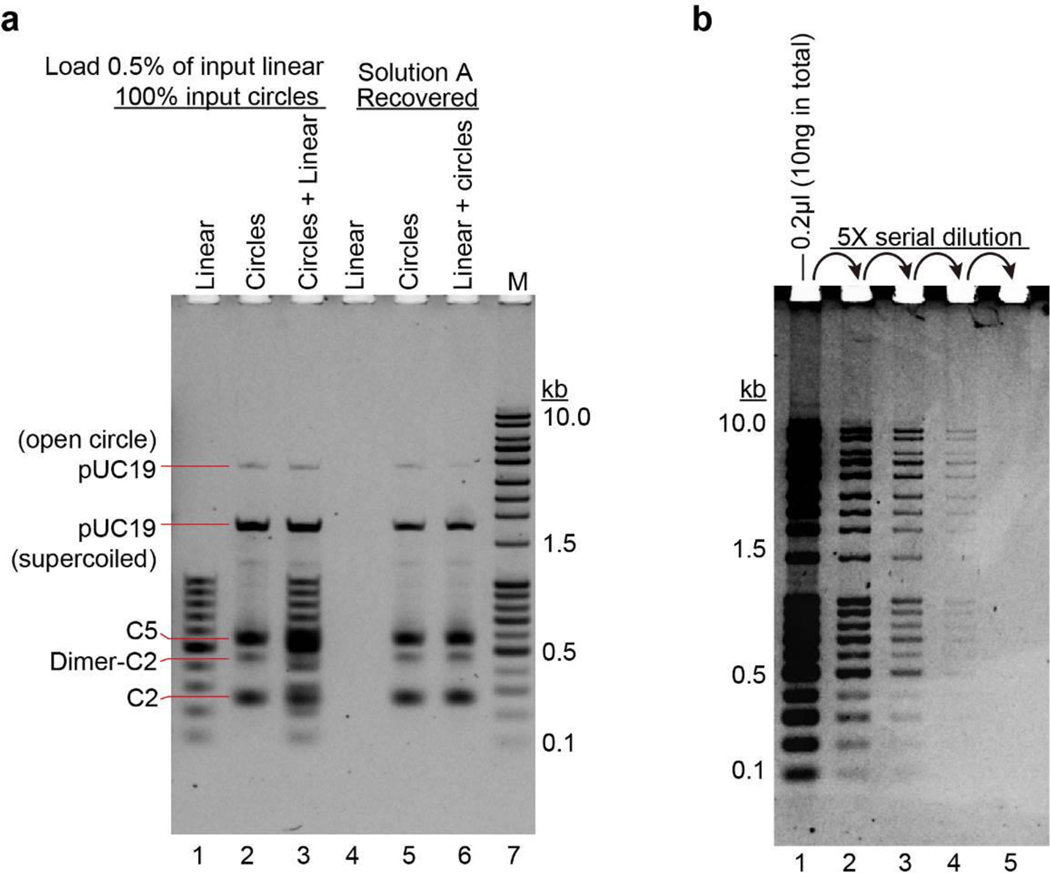
Selective binding of eccDNAs to beads and high sensitivity of DNA detection by SYBR Gold staining **a**, Representative gel image showing selective binding of circular DNA to magnetic silica beads in solution A. The assay was tested with 200× (mass) linear DNA (lane 4 and 6) and 1X (mass) DNA circles (lane 5 and 6). Lanes 1–3 indicate the input DNAs. Lane 1, 0.5% of input linear DNA for lane 4 was loaded; lane 2, equal amount of input circular DNAs for lane 5 were loaded; lane 3, 0.5% of input linear DNA and equal amount of input circular DNAs for lane 6 were loaded. Lanes 4–6 were DNAs recovered from Solution A. lane 4, 200× (mass) linear DNA alone undergone purification by solution A; lane 5, 1× (mass) DNA circles undergone purification by solution A; lane 6 mixture of 200× (mass) linear DNA and 1× (mass) DNA circles undergone purification by solution A. Note, only circular DNAs were recovered. The recovery rate is very high, particularly for the smaller circular DNAs. **b**, High sensitivity of DNA detection using vertical agarose gel electrophoresis and SYBR Gold staining. 0.2 μl commercial DNA ladder (total of 10 ng) was undergone 5× series dilutions and fractionated by 1% vertical agarose gel electrophoresis, stained by SYBR Gold, DNA in lane-4 were 125× diluted of that in lane-1, and could still be visualized. The detection limit is estimated as 5–10 pg/band. Results in **a–b** are representative of three independent experiments.

**Fig. 1.**
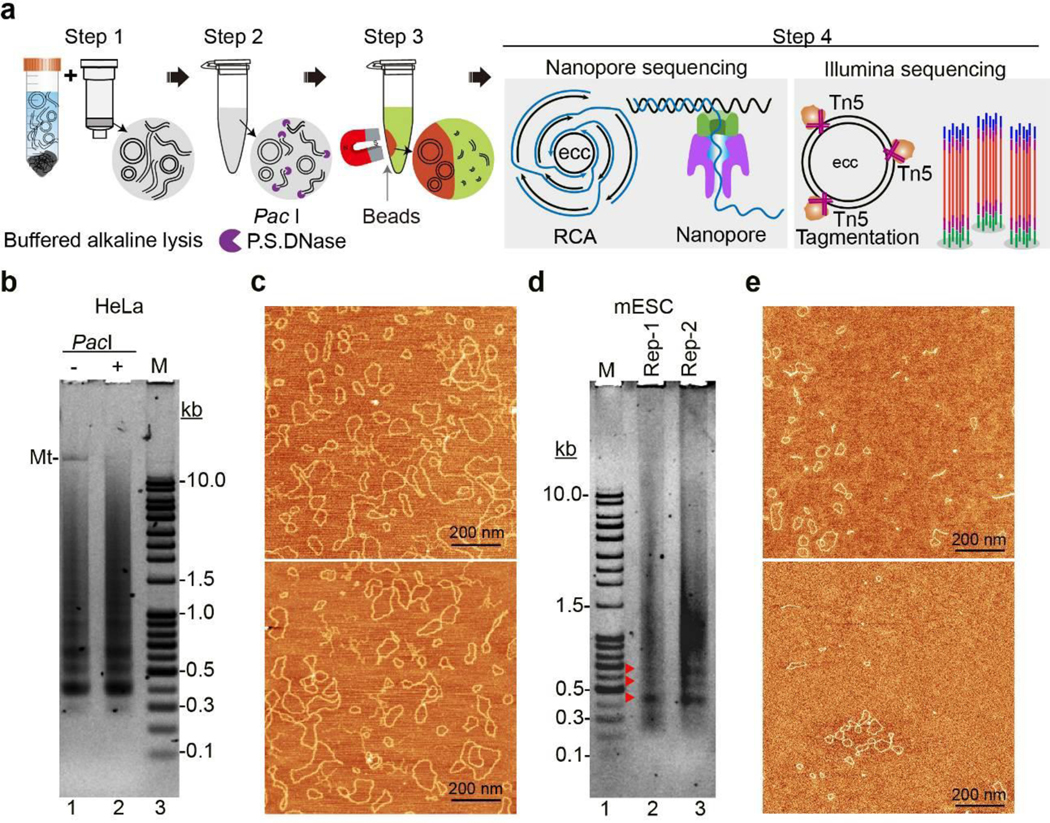
Development of a three-step eccDNA purification procedure **a**, Schematic of the three-step eccDNA purification and sequencing procedure. Step 1, extract crude DNA circles from whole cells in a buffered alkaline lysis and bound to silica column; Step 2, linearize mtDNA by *Pac*I and reduce overall linear DNA level with P.S. DNase; Step 3, Selectively recover eccDNAs by excluding residual linear DNA in Solution A; Step 4, sequence eccDNAs by Oxford Nanopore (left) following RCA or by Illumina after Tn5 tagmentation on eccDNAs (right). **b**, Agarose gel showing eccDNAs purified from over-confluent HeLa without (−) or with (+) *Pac*I treatment. Mt: mtDNA, M, linear DNA maker. **c**, EccDNAs in (**b**) scanned with atomic force microscope (SAFM). **d**, Agarose gel showing eccDNAs purified from normal cultured mESCs. Red arrow heads indicate distinct DNA bands. **e**, EccDNAs in (**d**) by SAFM. Representative gels are shown from three independent experiments (**b** and **d**); two representative fields are shown, black bars = 200 nm in (**c** and **e**).

We have developed a new three-step eccDNA enrichment method that allows efficient eccDNA purification (Fig. 1a). In the first step, to minimize eccDNA loss, we changed the unbuffered sodium hydroxide that can cause denaturation or breakage of DNA circles^11^ to modified alkaline lysis at pH 11.8. In the second step, we used a rare-cutter *Pac*I restriction enzyme to linearize mtDNA before an exonuclease (P.S. DNase, Plasmid safe ATP-dependent DNase) is used to digest linear DNA. In the third step, Solution A that could selectively recover circular DNA, but not linear DNA, on silica beads (Extended Data Fig. 1a) is used to exclude any linear DNA that has escaped the exonuclease digestion (Fig. 1a). Additionally, vertical agarose gel electrophoresis was used to increase the sensitivity of eccDNA detection (Extended Data Fig. 1b). Using this three-step purification procedure, we purified eccDNA from 10 million HeLa cells growing under confluence, a stress condition known to increase eccDNA abundance^12^. The purified eccDNAs exhibited discrete banded pattern (Fig. 1b). Furthermore, mtDNA could be removed by *Pac*I treatment (Fig. 1b, compare lanes 1 and 2). We further confirmed the circularity of the purified eccDNAs using scanning atomic force microscope (SAFM) (Fig. 1c). To determine whether eccDNAs occur in non-cancer cells, mouse embryonic stem cells (mESCs) were used, and the mESCs derived eccDNAs also exhibited a similar banded pattern (Fig. 1d), and their purity and circularity were also verified by SAFM (Fig. 1e).

## EccDNAs are mapped to the entire genome

**Extended Fig. 2.**
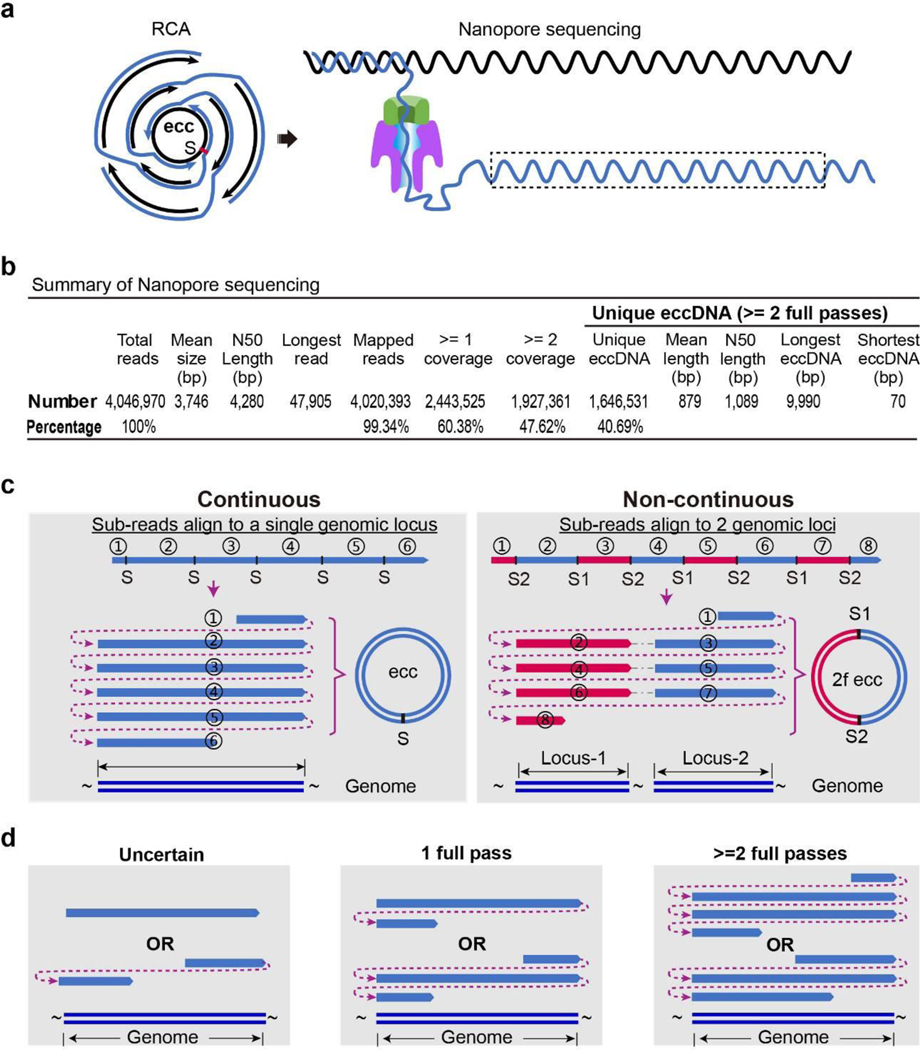
Summary of the nanopore sequencing data. **a**, Diagram of nanopore long read sequencing of eccDNA. Tandem copies of eccDNAs were self-concatenated to long molecule by rolling cycle amplification (RCA), and directly read through by Oxford Nanopore. Each copy of eccDNA molecule in a single long molecule was sequenced multiple times. “S”: split site. **b**, Summary of mESC eccDNA long reads from an Oxford Nanopore MinION flow cell. **c**, Diagram showing how the eccDNA full-length sequence is called and categorized. Nanopore reads from (**a**), the dashed box, were aligned to a single locus (Continuous) or to multiple loci (Non-continuous) in genome. Continuous: an example of six sub-regions of a long nanopore read repeatedly aligned to a single locus, where full length sequence of eccDNA was presented with only one split site (S); Non-continuous: an example of eight sub-regions of a single long read sequentially aligned to two separate loci (Locus-1 and Locus-2), representing an eccDNA ligated by two genomic fragments (2f ecc) with two split sites (S1 and S2). **d**, Criteria for eccDNA calling. EccDNAs were called based on their number of full passes aligned to the genome. Long reads with less than two full passes were discarded (left and middle panel). Left, Nanopore read that hits genome only once either fails to designate the genomic start and end site of eccDNA (up panel), or miss the middle region (bottom panel) that may or may not include in the original eccDNA molecule (Uncertain); Middle, because of potential sequencing error of Oxford Nanopore, reads that hit genome more than once but less than twice (1 full pass) were also discarded due to the lack of confirmation in eccDNA calling, particularly on designating the start and end site of eccDNA. Right, eccDNA molecules were called from long reads when covered at least twice (>=2 full pass) on their aligned loci.

**Fig. 2.**
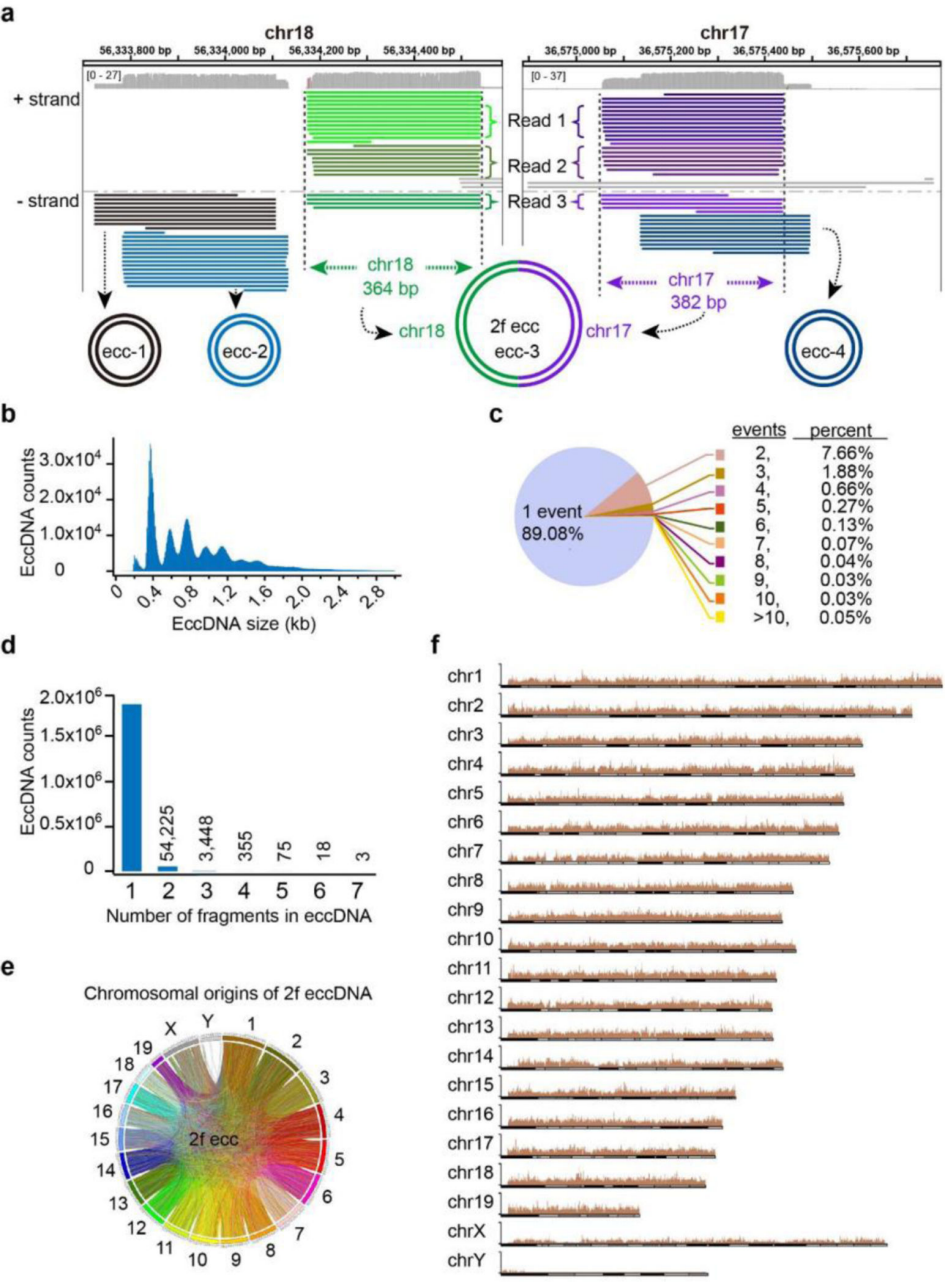
EccDNAs are circularized genomic DNA fragments that mapped across the genome **a**, Integrative Genomics Viewer (IGV) showing eccDNA examples from two genomic loci on chromosome 18 and 17. Individual horizontal bars in identical color represent sub-reads from a unique nanopore long read that was repeatedly aligned to the same genomic locus (loci). ecc-1, ecc-2 and ecc-4 are single-fragment circles; ecc-1 partially overlaps with ecc-2; ecc-4 partially overlaps with one fragment of ecc-3, is a two-fragment circle (2f ecc) aligned to two loci on chr18 and chr17, respectively. **b**, Histogram showing the eccDNA size distribution and relative abundance. **c**, Pie chart showing the percentages of eccDNA with the indicated event number in the total unique eccDNAs identified. **d**, Bar graph showing eccDNA counts with the number of fragments (1–7) in each circle. **e**, Circle plot showing chromosomal origin of all 2 fragments eccDNAs (2f ecc). Fragments from the same chromosome are in the same color. **f**, Overall chromosomal distribution of eccDNAs across the genome.

To gain insights into the potential mechanism of eccDNA biogenesis, we determined the genomic source of eccDNAs. HeLa cells are notorious for their aberrant genome, including aneuploidy and numerous structural variations, such as deletions, duplications, inversions, translocations and rearrangements etc.^13^, making interpretation of sequencing data and dissection of the eccDNA biogenesis mechanism difficult. Thus, we performed eccDNA sequencing and mapping using mESCs, whose genetic integrity is maintained during culture^14^. To obtain full-length eccDNA sequences, we performed RCA amplification and subsequent long read Nanopore sequencing of the multiple tandem copies of individual eccDNA molecule (Fig.1a, Extended Data Fig.2a). The repeated sequencing of the same eccDNA multiple times of long reads allows the generation of a consensus sequence that matches the full-length sequence of the original eccDNA by a computational threading method (Fig. 2a **and** Extended Data Fig. 2c). We obtained 4 million long reads with a mean size of 3.7 kb (Extended Data Fig. 2b). To reduce false positives due to RCA artifacts and sequencing errors of Nanopore technique^15^, we only used the 1.9 million long-reads with each of them contains at least two full passes on the same eccDNA to identify high confidence eccDNAs, resulting the identification of 1.6 million unique eccDNAs with a median size of 1 kb (Extended Data Fig. 2b). Interestingly, the eccDNAs exhibit a regular 188 bp average size interval (Fig. 2b), and the great majority (89%) of unique eccDNAs were sequenced from a single long read (single-event eccDNA), and less than 1.5% of unique eccDNAs were sequenced from more than three unique long molecules (Fig. 2c) and no dominant eccDNA was identified. Such large numbers of single-event eccDNAs coupled with the lack of dominant eccDNA molecules suggest that the eccDNAs are unlikely derived from specific genome regions.

Genome mapping of full length eccDNA revealed their various genomic alignment patterns, including adjacent, overlapped, nested, or even across different chromosomes (Fig. 2a). We found that a great majority of eccDNAs were originated from single continuous genomic locus (Continuous eccDNA), and only a relatively small number of eccDNAs were formed by multiple genomic fragments (Non-continuous eccDNA) (Fig. 2d, Extended Data Fig. 2c), including 3 eccDNAs with 7 genomic fragments joined together to form a circle (7f eccDNA) (Fig. 2d). To determine whether the physical distance of genomic fragments affects the frequency of eccDNA formation, we analyzed genomic origin of 2 fragments eccDNAs (2f eccDNA). A circle plot clearly showed that paired fragments of 2f eccDNAs are not restricted to the same chromosome (Fig. 2e), but rather randomly bridged between chromosomes, indicating eccDNAs can be formed by joining genomic fragments from different chromosomes. Consistently, genome mapping of all eccDNAs revealed that eccDNAs are widespread across the entire genome (Fig. 2f).

**Extended Data Fig. 3.**
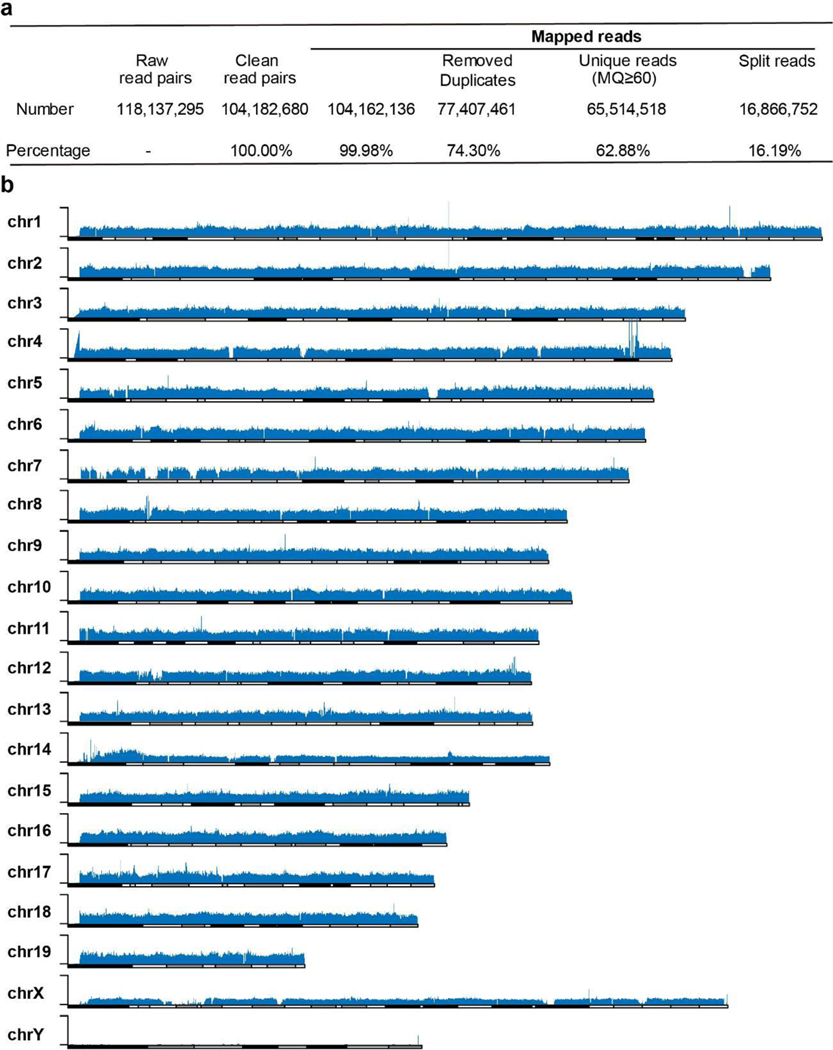
Summary of Illumina short read sequencing and Chromosomal distribution of eccDNA fragments. **a**, Summary of eccDNA short read sequencing without RCA. Purified eccDNA was directly tagmented with Tn5 (Illumina Nextera), and sequenced with Illumina Hiseq 2500 in PE150 mode. **b**, Chromosomal distribution of eccDNA short reads.

To rule out potential biases caused by uneven amplification by RCA^16^, we purified another batch of eccDNAs and directly tagged them with Tn5 transposase without RCA for Illumina sequencing (Fig.1a **and** Extended Data Fig. 3a). EccDNA sequences obtained this way should faithfully reveal their genomic location and relative abundance. Consistent with the Nanopore sequencing results, Illumina sequencing revealed widespread alignment of eccDNAs across the entire genome (Extended Data Fig. 3b). We noticed that the eccDNA density on X chromosome was about half of the autosomes (Fig. 2f **and** Extended Data Fig. 3b), consistent with the fact that diploid male genome of mESC/E14 cells carry one copy of X chromosome but two copies of autosomes (Fig.2f **and** Extended Data Fig.3b). The lack of eccDNAs mapped to the Y chromosome is largely due to the many undetermined sequences and repeat sequences in Y chromosome^17^. Collectively, these data suggest that eccDNAs are widespread across the entire genome and their abundances are correlated with genomic copy numbers.

## DNase γ is required for eccDNA generation

**Extended Data Fig. 4.**
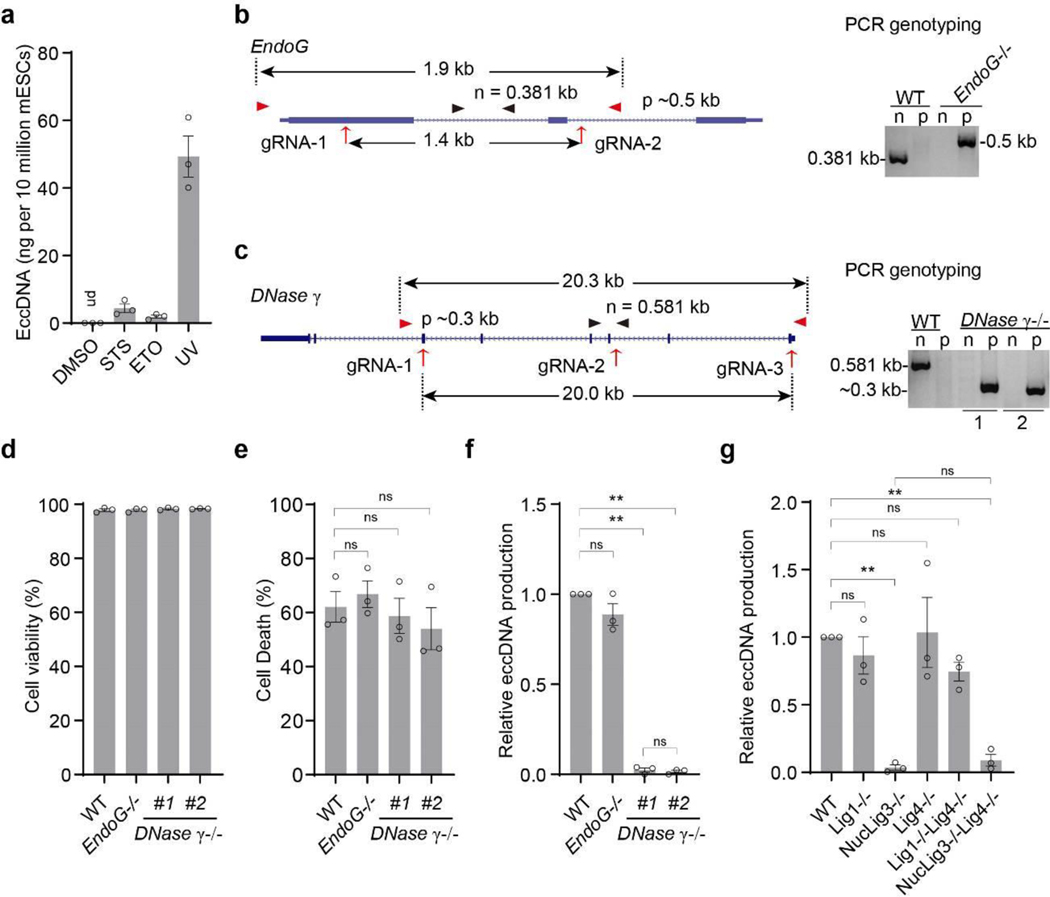
Effects of *Endonuclease G, DNase γ*, and DNA ligases knockout on apoptosis and eccDNA generation **a**, Quantification of eccDNA from cells of the indicated treatment. EccDNA production were presented as nanogram per 10 million cells; ud, under detection limit. Bars indicate mean ± SEM of three independent experiments. **b**, Diagram illustration and PCR confirmation of the *EndoG* knockout mESC line. Gene structure of *EndoG*, sgRNAs (red arrows), two sets of screen primers for internal sites (black arrowhead, “n” =negative knockout cell line) and external sites (red arrowhead, “p” =positive knockout cell line) were shown. **c**, Diagram illustration and PCR confirmation of the *DNase γ* knockout mESC lines. Gene structure of *DNase γ*, sgRNAs (red arrows), two sets of screen primers for internal sites (black arrowhead, “n” =negative knockout cell line) and external sites (red arrowhead, “p” =positive knockout cell line) were shown. **d**, Cell viability is not affected by *EndoG* or *DNase γ* KO. Cell viability was evaluated by flow cytometry, after staining with Far Red Live/Dead Cell Stain Kit, FSC-A/SSC-A and FSC-A/FSC-H were sequentially used to exclude debris and gate on singlets, respectively, then gate on APC+ as dead cells. Bars indicate mean ± SEM. of dead cell ratio from three independent experiments. **e**, Deficiency of *DNase 𝛾* or *EndoG* in mESC do not significantly alter UV-induced cell death. Cell death was measured by flow cytometry, after staining with Far Red Live/Dead Cell Stain Kit, FSC-A/SSC-A and FSC-A/FSC-H were sequentially used to exclude debris and gate on singlets, respectively, then gate on APC+ as dead cells. Bar indicate mean ± SEM. of dead cell ratio of 3 independent experiments. **f**, Quantification of eccDNAs presented in Fig. 3d. EccDNAs below the mtDNA band were quantified by densitometry (by Image J 1.53e) and presented as relative levels to that in WT cells of parallel eccDNA purifications. Bars indicate mean ± SEM. of three independent experiments. **g**, Quantification of eccDNA below the mtDNA band by densitometry (by Image J 1.53e). Data is presented as relative levels to that purified from WT cells of parallel eccDNA purifications (Fig. 3g), bars indicate mean ± SEM of three independent experiments. Ordinary one-way ANOVA with Tukey’s multiple comparison test was used for multiple comparisons in **e**, **f**, **g**. **, *p*<0.01; ns, no significant.

**Fig. 3.**
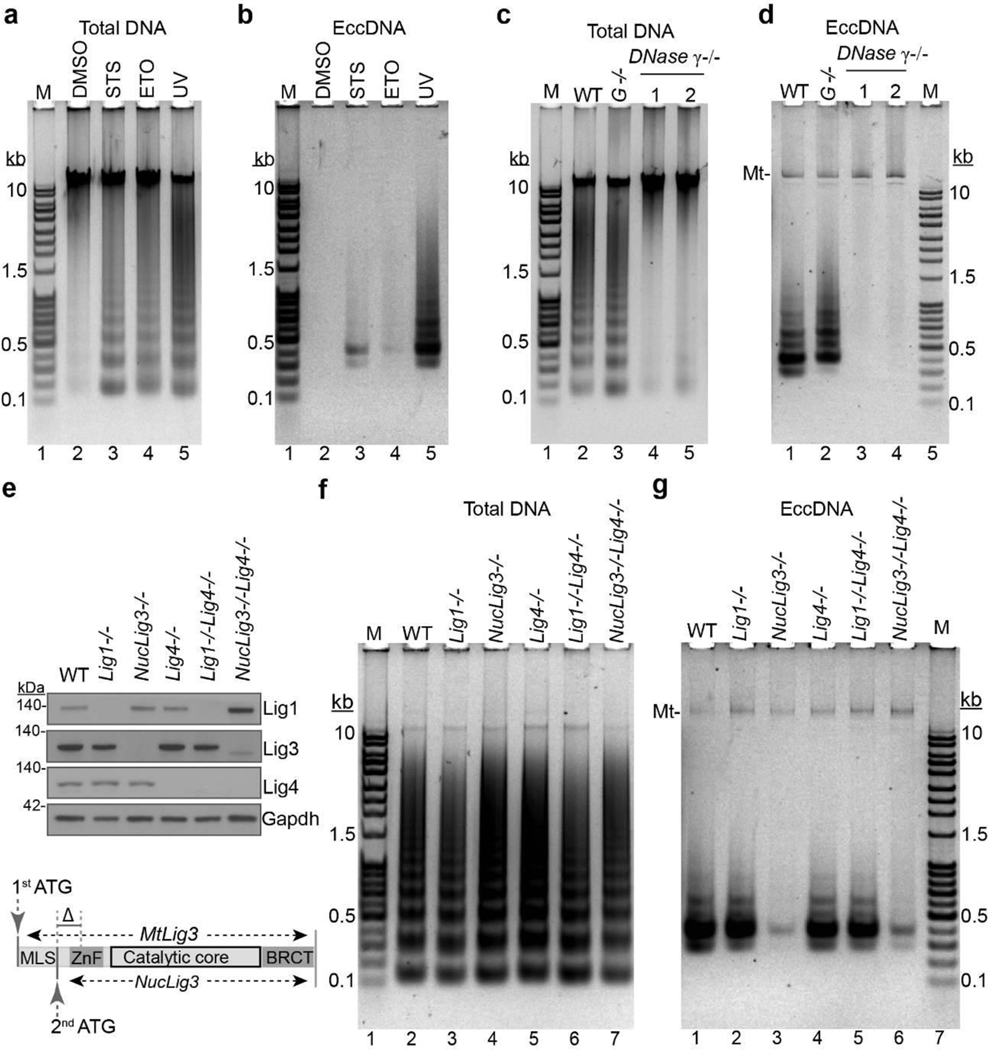
Apoptotic DNA fragmentation and subsequent ligation by Lig3 is required for eccDNA production in mESCs **a-b**, EccDNA production is induced by apoptosis. mESCs were treated with the indicated apoptosis inducers, and total DNA (**a**) and eccDNAs (**b**) were purified. DMSO: Dimethylsulfoxide; STS: Staurosporine; ETO: etoposide; UV: ultraviolet. **c-d**, Deficiency of oligonucleosomal DNA fragmentation abolishes apoptosis induced eccDNA production. Deficiency of *DNase 𝛾*^−/−^, but not *EndoG*^−/−^, abolishes UV-induced oligonucleosomal DNA fragmentation (**c**), and eccDNA production (**d**) in mESCs. MtDNA was kept as an inner control. **e**, Confirmation of DNA ligases deficient cell lines. Up, immunoblotting confirming knockout of DNA ligases in CH12F3 cell lines. Bottom, genomic structure of *Lig3* with CRISPR/Cas9 specific targeting (Δ) *NucLig3* but retaining *MtLig3* for cell viability. **f-g**, *Lig3* is the major DNA ligase for eccDNA generation. Staurosporine induced oligonucleosomal DNA fragmentation (**f**) and eccDNA (**g**) from the indicated CH12F3 cell lines. Amount of input cells, elution and loading volume were equal among samples within each agarose gel (**a-d**, and **f-g**). Shown are representatives of three independent experiments.

The great diversity, randomness, and the nucleosome “ladder” size (Fig. 2) suggest that eccDNAs might be generated by random ligation (including self-ligation) of oligonucleosomal DNA fragments, which can be visualized as “ladders” in agarose gel, and is a known feature of apoptosis^24^. To determine if apoptotic cells are the source of eccDNAs, mESCs were treated with apoptosis inducers, staurosporine (STS), etoposide (ETO) or UV-light. Successful induction of apoptosis was confirmed by the typical nucleosomal “ladder” pattern of genomic DNA (Fig. 3a). When equal amount of control (DMSO) and treated cells were subjected to the 3-step eccDNA purification procedure and visualized on an agarose gel, all the three treatments induced eccDNA production although UV treatment has the strongest induction (Fig. 3b, Extended Data Fig. 4a).

We next determined if eccDNA generation requires apoptotic DNA fragmentation (ADF), which is mediated by Caspase activated DNase (CAD)^18^, Endonuclease G (EndoG)^19^ or DNase γ^20^ in a cell type-specific manner. Genetic manipulation (Extended Fig. 4b, c) indicated that DNase γ, but not EndoG, mediates ADF in mESC, as indicated in the lack of “ladder” pattern^20^ (Fig. 3c). DNase γ knockout does not affect cell viability under either normal culture conditions or UV treatment (Extended Data Fig. 4d, e). Purification of the eccDNAs from UV-treated cells demonstrated that abrogation of ADF prevented eccDNA generation (Fig. 3d, Extended Data Fig. 4f). We intentionally skipped *Pac*I digestion to retain mtDNA as an internal control for equal cell input and circular DNA recovery (Fig. 3d). These results demonstrate that ADF is a prerequisite for eccDNA generation.

## Lig3 is required for eccDNA generation

**Fig. 4.**
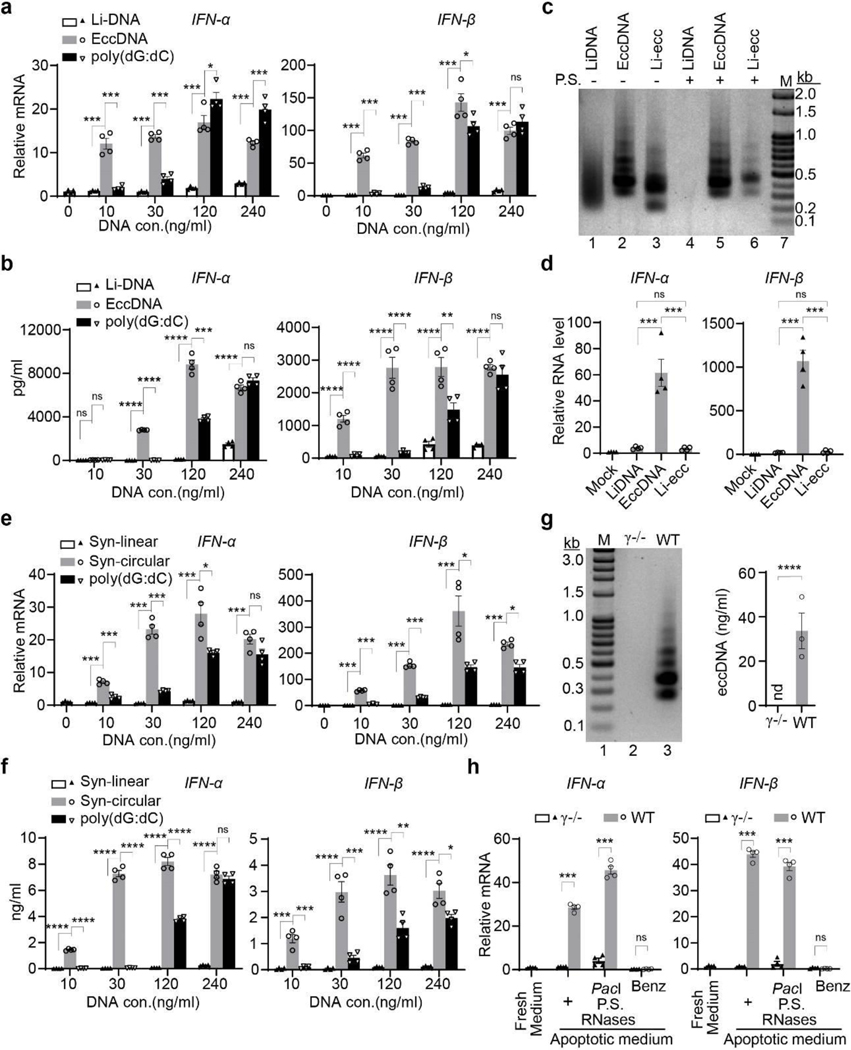
EccDNAs are potent immunostimulants **a**, EccDNAs induce *IFN-α* and *IFN-β* expression in BMDCs. Equal amount of the indicated DNA were transfected to BMDC at increasing concentrations for RT-qPCR analysis. Data are presented as relative mRNA fold change (y-axis) to that in mock transfection (without DNA). Li-DNA: sonicated genomic linear DNA with sizes similar to that of eccDNAs; poly(dG:dC), poly(deoxyguanylic-deoxycytidylic). **b**, ELISA analysis of *IFN-α* and *IFN-β* production in medium from (**a**). **c**, Confirming eccDNA linearization. Representative gel shows equal amount of the indicated DNA digested with or without P.S. DNase (P.S.). Li-ecc: linearized eccDNA. **d**, Linearized eccDNAs lost their immunostimulatory activities. DNA of lanes 1–3 from (**c**) were transfected to BMDCs at 30 ng/ml and mRNAs were evaluated as in (**a**). **e-f**, Synthetic small DNA circles are potent immunostimulants. The same experiments were performed as in (**a-b**) except the li-DNA and eccDNA were replaced by the synthetic linear (Syn-linear) and circular (Syn-circular) DNAs of the same sequences, mRNA (**e**) and ELISA (**f**). **g**, EccDNAs are present in supernatant of apoptotic medium of WT, but not of *DNase γ*−/− cells. Representative gel of eccDNA (left) and quantifications (right, n=3); nd, not detected. **h**, Exonuclease resistant DNA (not mtDNA) in the supernatant of apoptotic medium activate *IFN-α* and *IFN-β*. Supernatants of apoptotic medium from WT or *DNase γ*−/− cells were treated as indicated, then incubated with BMDCs for RT-qPCR analysis as in (**a**). *Pac*I (for linearizing mtDNA), Benz: Benzonase. Representative data were shown as mean ± SEM of replicates (n=4 per group in **a-b**, **d-f**, and **h**;) of three independent experiments. Statistics were calculated on biological replicates with ordinary one-way ANOVA with Tukey’s multiple comparison test (**a-b**, **d-f**, and **h**) or two-tailed unpaired t tests (**g**). *, *p*<0.05; **, *p*<0.01; ***, *p*<0.001; ****, *p*<0.0001; ns, no significant.

Next, we attempted to identify the DNA ligase responsible for circularizing the fragmented DNA. Mammals have three DNA ligase genes (*Lig1*, *3*, and *4*) each with specific function and they also function redundantly in DNA metabolism^21^. The functions of these ligases have been well studied in the CH12F3 mouse B lymphocyte cell line^22^. To determine which of the three DNA ligases is responsible for the ADF circularization, individual DNA ligase and their combinations were knocked out in CH12F3 cell by the CRISPR/Cas9 technique and confirmed by Western blotting (Fig. 3e). *Lig3* has both nuclear and mitochondrial isoforms, and the later isoform is essential for mitochondria maintenance and consequently cell viability^23^. Thus, the *Lig3* knockout cell line was generated by specifically targeting the nuclear isoform (*NucLig3*−/−) without interfering the mitochondrial isoform (*MtLig3*, Fig. 4a, lower panel). Equal number of WT (wildtype) and mutant cells were treated with staurosporine to induce apoptotic DNA fragmentation (Fig. 3f), and eccDNAs were purified and visualized in agarose gel (Fig. 3g). The results indicated that knockout *Lig1* or *Lig4* alone or in combination did not significantly affect eccDNA generation. In contrast, knockout of *Lig3* greatly reduced eccDNA generation (Fig. 3g, Extended Data Fig. 4g). Since *Lig1* and *Lig3* double knockout is cell lethal^22,23^, it is unknown whether the double knockout can completely abrogate eccDNA generation. Nevertheless, these data supports *Lig3* as the main ligase for eccDNA generation in CH12F3 cells.

## EccDNAs are potent innate immunostimulants

**Extended Data Fig. 5.**
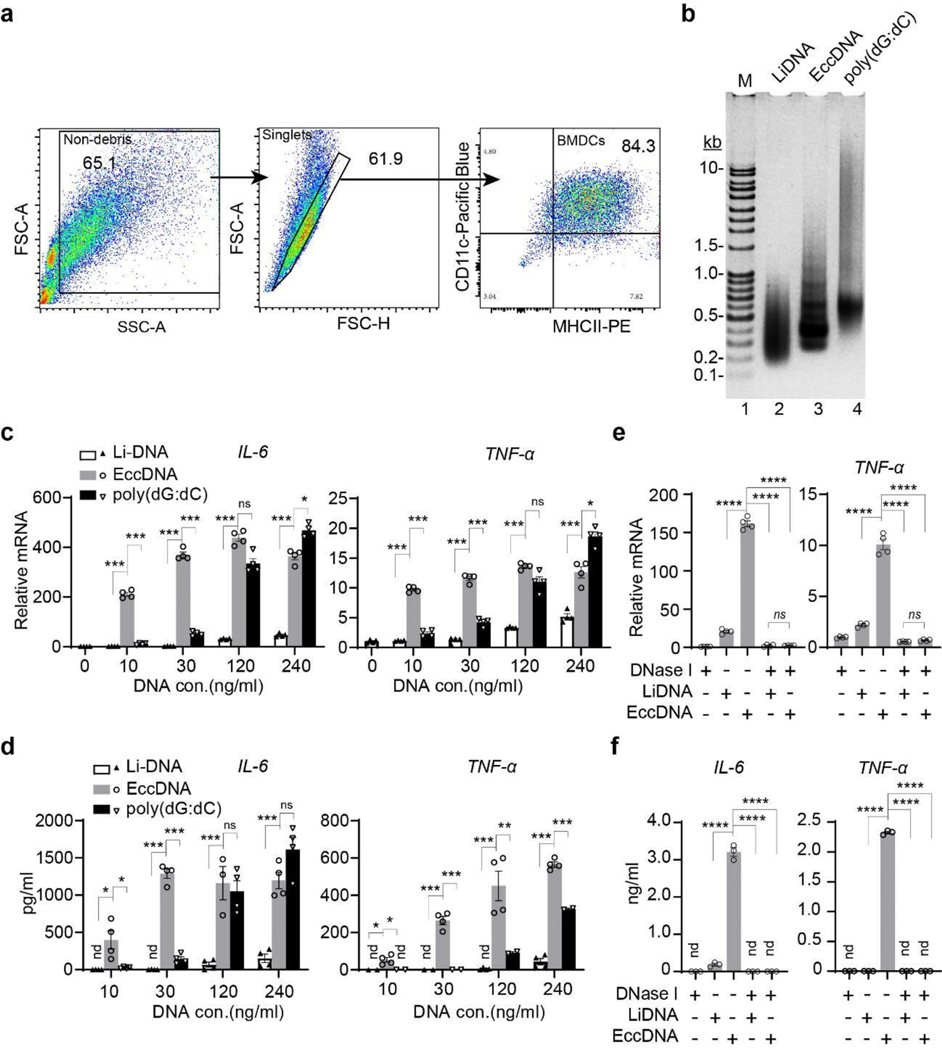
EccDNAs are potent immunostimulants for BMDCs **a**, Confirmation of BMDC identity by flow cytometry. After differentiating bone marrow cells with 20 ng/ml GM-CSF for 7 days, cell debris (left) were first excluded and singlets (middle) were then phenotyped on the basis of their CD11c and major histocompatibility complex II (MHCII) expression (right) to define BMDC, the numbers indicate the percentage of gated cells. Data were further analyzed with FlowJo. V10.8.0 and shown as representative of 2 independent experiments. **b**, Various DNAs were resolved by agarose gel electrophoresis. Li-DNA: sheared linear genomic DNA; poly(dG:dC), poly(deoxyguanylic-deoxycytidylic). Representative gel of three independent experiments was shown. **c-d**, Bar graphs showing the relative mRNA (**c**) and protein (ELISA) (**d**) levels of *IL-6* and *TNF-α*. Data is shown as mean ± SEM of replicates (n=4 per group) of a representative experiment in three independent experiments. **e-f**, DNase I pretreatment abolishes *IL-6* and *TNF-α* induction by eccDNA. Equal amount of DNA (120 ng/ml) used to prepare transfection mixtures were pre-treated with or without DNase I as indicated, then transfected to BMDCs. 12 hours later, total RNA was extracted for RT-qPCR (**e**) and medium was collected for ELISA (**f**). Data is shown as mean ± SEM of replicates (n=4 per group) of a representative experiment in three independent experiments. Comparisons in **c-f** were done on biological replicates by Ordinary one-way ANOVA with Tukey’s multiple comparison test. *, *p*<0.05; **, *p*<0.01; ***, *p*<0.001; ****, *p*<0.0001; ns, no significant; nd, not detected.

The above results demonstrate that eccDNAs are ligation products of fragmented genomic DNA of apoptotic cells. DNA released from dying cells has been previously reported to promote immune responses^24^. Two important mediators of immune response, Toll-like receptor 9 (TLR9)^25^ and high mobility group box 1 (HMGB1)^26^ have been reported to preferentially bind to DNA curvatures. These observations suggest that eccDNAs may serve as immunostimulants. To test this idea, we generated bone marrow derived dendritic cells (BMDCs) (Extended Data Fig. 5a), and compared the immunostimulatant activity of sheared linear genomic DNA (Li-DNA), eccDNAs, and the widely used potent DNA ligand of cytosolic DNA sensors, poly(dG:dC)^27^ (Extended Data Fig. 5b). We transfected BMDCs with different amount of the three forms of DNAs and then collected cells for quantitative reverse transcription polymerase chain reaction (RT-qPCR) assays. Results show that, compared to linear DNA transfection, type I interferons (IFN-α, IFN-β), interleukin IL-6, and tumor necrosis factor (TNF-α) were all significantly induced by eccDNAs at a wide range of concentrations (10–240 ng/ml) (Fig. 4a, Extended Data Fig. 5c). Surprisingly, the widely regarded “potent” cytokine inducer poly(dG:dC) is not as nearly potent as eccDNAs at lower concentrations, and the linear DNA only triggered a mild response even at the highest concentration when compared to mock transfection, indicating that dendritic cells are much more sensitive to eccDNA treatment than that of linear DNA and poly(dG:dC). Consistently, enzyme-linked immunosorbent assay (ELISA) confirmed the strong potency of eccDNAs in cytokine induction (Fig. 4b, Extended Data Fig. 5d).

**Extended Data Fig. 6.**
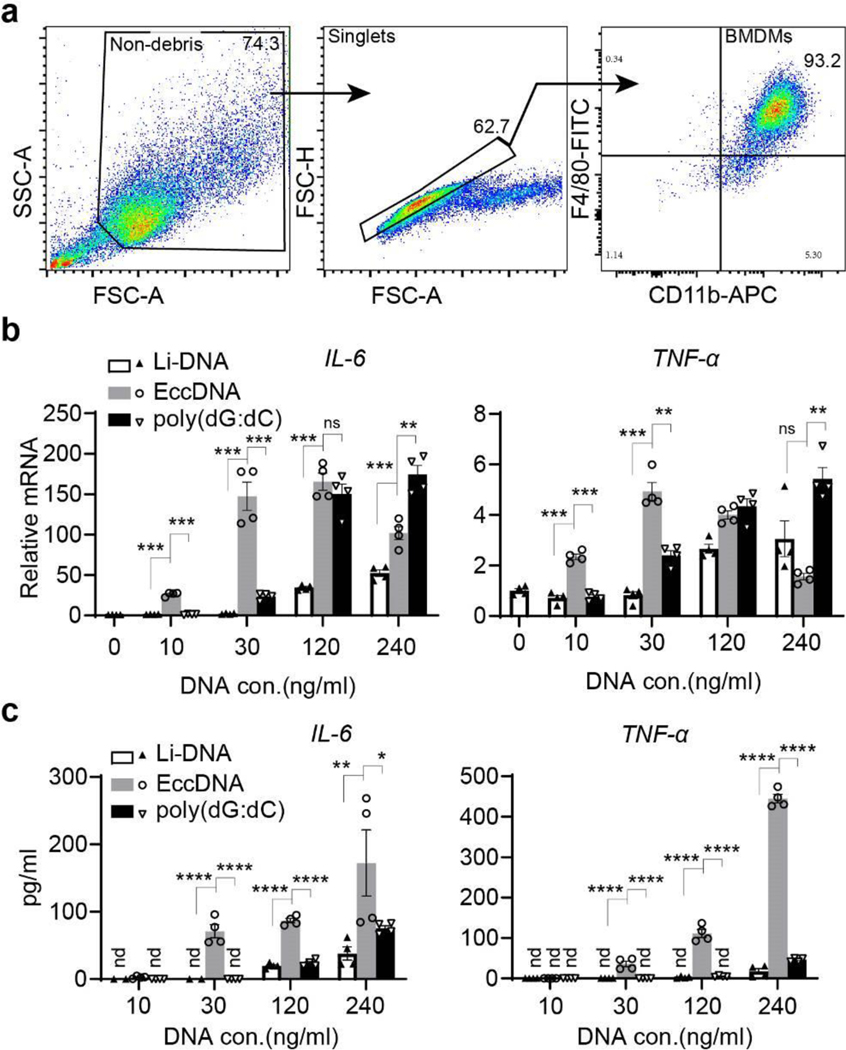
EccDNAs are potent immunostimulants for BMDMs **a**, Confirmation of BMDMs identify by flow cytometry. After differentiating bone marrow cells with L929 conditioned medium for 7 days, cells debris (left) were first excluded and singlets (middle) then phenotyped on the basis of F4/80 and CD11b expression (right) to define BMDM, the numbers indicate the percentage of gated cells. Data were further analyzed with FlowJo. V10.8.0 and shown as representative of 2 independent experiments. **b-c**, EccDNAs induce cytokine genes in BMDMs. Bar graphs showing the induction of mRNA (**b**) and protein (**c**) of *IL-6* and *TNF-α* in BMDMs that transfected with varying levels of eccDNA, compared with fragmented linear DNA, and poly(dG:dC). RT-qPCR were performed after 12-hours transfection. ELISA analysis (**c**) was performed after 24-hours transfection. Data is shown as mean ± S EM of replicates (n=4 per group) of a representative experiment of three independent experiments. Comparisons were performed on biological replicates with equal amount of DNA transfection by Ordinary one-way ANOVA with Tukey’s multiple comparison test. *, *p*<0.05; **, *p*<0.01; ***, *p*<0.001; ****, *p*<0.0001; ns, no significant; nd, not detected.

In addition to dendritic cells, macrophages are also known to respond to immunostimulants^28^. To determine whether macrophages behave like dendritic cells upon eccDNA transfection, we generated bone marrow derived macrophages (BMDMs) (Extended Data Fig. 6a). Similar to that observed in BMDCs, eccDNAs also displayed much higher immunostimulant activities in BMDMs than li-DNA or poly(dG:dC), particularly at lower concentrations (10, 30 ng/ml)(Extended Data Fig. 6b, c). These data indicate that eccDNAs are very potent immunostimulants in activating both BMDCs and BMDMs. Furthermore, pretreatment of eccDNA with DNase I before transfection completely abrogated the capacity of eccDNA to induce cytokine production (Extended Data Fig. 5e, f), demonstrating eccDNA, rather than potential concomitants of eccDNA, is responsible for the immune activation.

## Circularization confers eccDNA’s potency

**Extended Data Fig. 7.**
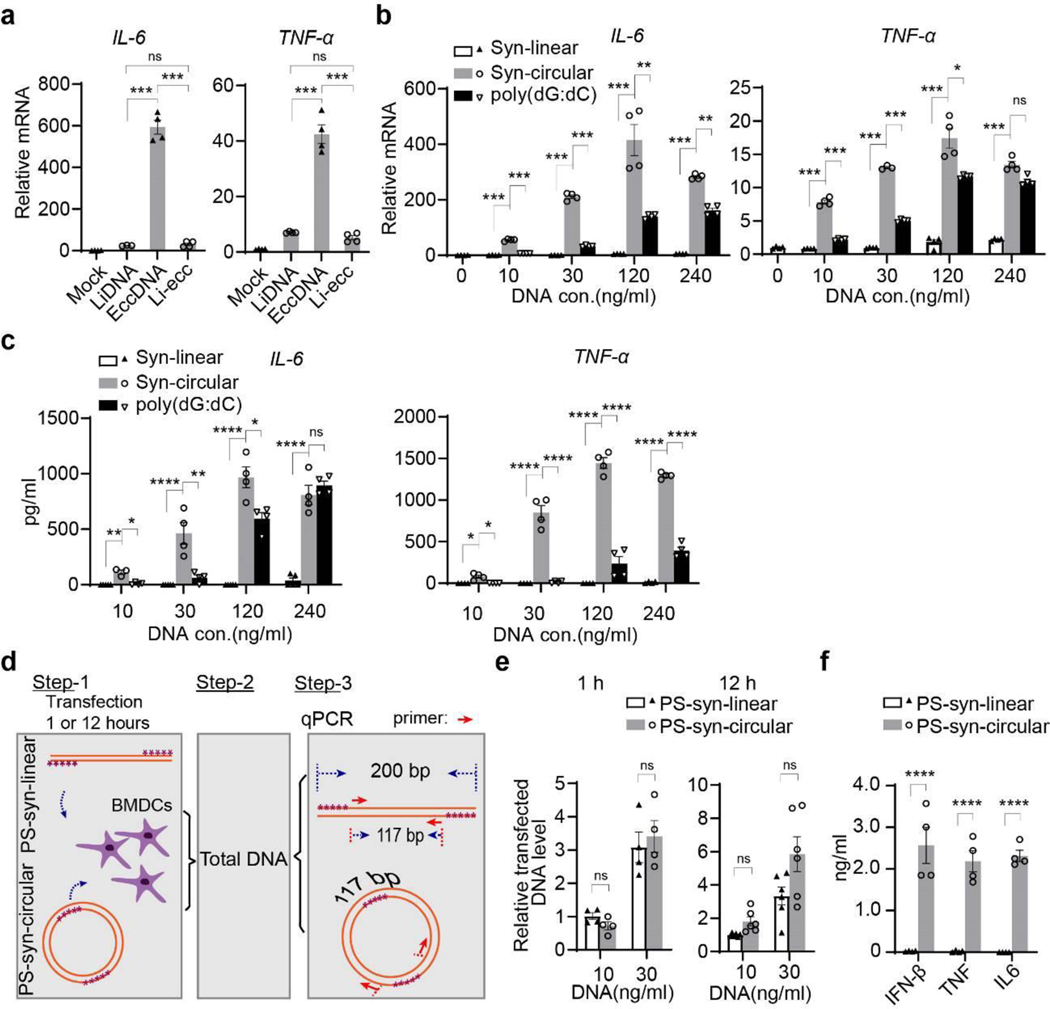
The circularity of eccDNAs, but not the sequence, is critical for their immunostimulant activity **a**, Bar graphs showing the relative *IL-6* and *TNF-α* mRNA levels. Data is shown as mean ± S EM of replicates (n=4 per group) of a representative experiment in three independent experiments. **b-c**, Bar graphs showing the relative *IL-6* and *TNF-α* mRNA levels (**b**), and protein (ELISA) levels (**c**). Data is shown as mean ± SEM of replicates (n=4 per group) of a representative experiment in three independent experiments. **d**, Diagram of DNA transfection efficiency and stability assay. Step-1: 5 Phosphorothioate (purple “*”) end-protected synthetic linear DNA (PS-syn-linear) and its circular form with equal number of phosphorothioate bonds (PS-Syn-circular) were transfected to BMDCs in the same way as in Fig. 5 for either 1 or 12 hours. Step-2: cells were lysed in 100 μl lysis buffer and treated with Thermoliable Proteinase K to prepare total DNA. Step-3: 4 μl total DNA was used for qPCR with a set of primers that amplify a 117 bp fragment in both linear and circular DNA. **e**, qPCR analysis of the samples prepared as described in (**d**). Transfected DNA level was normalized to that of 10 ng/ml PS-syn-linear transfection (n= 4 or 6, as indicated by dotes). Bars indicate mean ± SEM. of three independent experiments. **f**, 12 hours after transfection of indicated DNA at 30 ng/ml, medium was collected for ELISA essay, Data is shown as mean ± SEM of replicates (n=4 per group) of a representative experiment in three independent experiments. Comparisons in **a-c** were performed on biological replicates by Ordinary one-way ANOVA with Tukey’s multiple comparison test; two-tailed unpaired *t* tests (**e-f**). *, *p*<0.05; **, *p*<0.01; ***, *p*<0.001; ****, *p*<0.0001; ns, no significant.

To determine whether the circular nature of eccDNAs is critical for their strong immunostimulatory activity, purified eccDNAs were first treated with FnoCas12a (Cpf1), which introduces one nick per circular DNA in the presence of Mn^2+^, and the absence of guide RNA^29^. The nicked eccDNAs were then treated with single strand specific endonuclease Nuclease S1, which cleaves the intact circular strand at the site opposite to the nick to generate linearized eccDNA (Li-eccDNA). The linearization of eccDNAs was confirmed by their sensitivity to exonuclease digestion, while intact eccDNAs were resistant (Fig. 4c, **compare lanes** 5 **and 6**). When equal amount of linear DNAs, eccDNAs, and Li-eccDNAs were transfected to BMDCs, Li-eccDNAs behaved like linear DNAs and failed to activate IFN-α, IFN-β, IL-6, or TNF-α (Fig. 4d, Extended Data Fig. 7a). These results demonstrated that the circular nature of eccDNAs is critical for their strong immunostimulant activity. Since eccDNAs are derived from randomly ligated genomic fragments, their sequences are unlikely to significantly contribute to their potency. This notion was confirmed by the demonstration that a synthetic 200 bp circular DNA, but not its linear counterpart, greatly induced cytokine gene transcription in BMDCs (Fig. 4e, Extended Data Fig. 7b). Similar to native eccDNAs, synthetic circular DNA also showed higher potency in cytokine gene activation when compared to that of poly(dG:dC) (Fig. 4e, Extended Data Fig. 7b). Consistently, ELISA confirmed the strong cytokine induction capacity of synthetic circular DNA (Fig. 4f, Extended Data Fig. 7c).

To rule out the possibility that the increased immunostimulatory potency of circular DNA is due to its increased stability and transfection efficiency, and to minimize the effects of exonuclease activity on linear DNA, we added phosphorothioate^30^ bonds on both ends of the 200 bp linear DNA (PS-Syn-linear). To exclude the potential effect of phosphorothioate bonds on transfection and immune stimulation, equal number of phosphorothioate bonds were also put in the circular counterpart (PS-Syn-circular). Both linear and circular DNAs were separately transfected to BMDCs, cell lysates and culture media were collected for qPCR and ELISA assay to compare their transfection efficiency (1 hour after transfection), stability and cytokine induction (12 hours after transfection) (Extended Data Fig. 7d). We found no significant difference in their transfection efficiency or stability between the 200 bp linear and circular DNAs (Extended Data Fig. 7e). Yet, circular DNA induced high level of cytokine production while its linear counterpart did not (Extended Data Fig. 7f). Collectively, these data support that the circularity but not the sequence of eccDNAs confers the high potency of their immunostimulant activity.

## BMDC senses eccDNAs in apoptotic medium

Since eccDNAs are generated in apoptotic cells, they could be released into the culture medium. Indeed, significant amount of eccDNAs can be detected in cell free supernatant of apoptotic medium of UV treated mESCs (Fig. 4g). To determine if eccDNAs from the supernatant of apoptotic medium can be actively sensed by BMDCs without transfection, BMDCs were co-incubated with cell-free apoptotic supernatant of WT or DNase γ−/− mESCs. RT-qPCR analysis indicates that the supernatant of apoptotic medium of WT, but not DNase γ−/− cells, stimulated IFN-α, and IFN-β expression (Fig. 4h). Importantly, this stimulation is not sensitive to pre-treatment of the supernatant with P.S. DNase (linear DNA-specific), *Pac*I restriction enzyme, and RNases that digest linear DNA, mitochondrial DNA, and RNA, respectively (Fig. 4h), but is sensitive to pre-treatment of Benzonase, a nuclease that destroys all forms of DNA and RNA without proteolytic activity (Fig. 4h). These data indicate that eccDNAs, but not linear DNAs, mitochondrial DNAs, or RNAs in the supernatant of apoptotic medium, are responsible for the induced immune response. Furthermore, this result also indicates that eccDNAs can be actively sensed by BMDCs without transfection. Collectively, our results indicate that eccDNAs are potent damage-associated molecular patterns (DAMPs) of the innate immune system^28^.

## eccDNA-triggered immune response requires Sting

**Extended Data Fig. 8.**
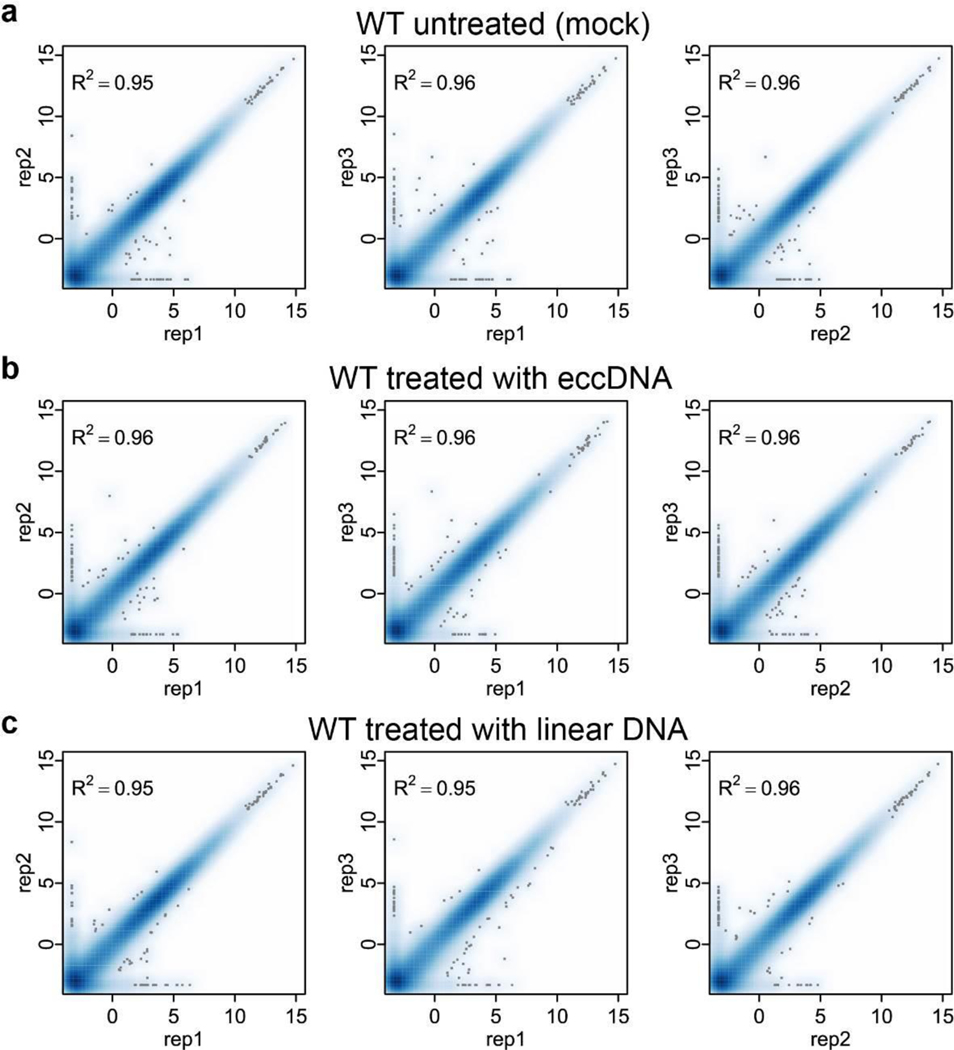
Reproducibility of the BMDC transcriptomes **a-c**, Scatter plot of pair-wise comparison of the transcriptomes of three independent BMDC samples of untreated (a), treated with eccDNA (b) or linear DNA (c). The x and y-axis are log2(FPKM+0.1).

**Extended Data Fig. 9.**
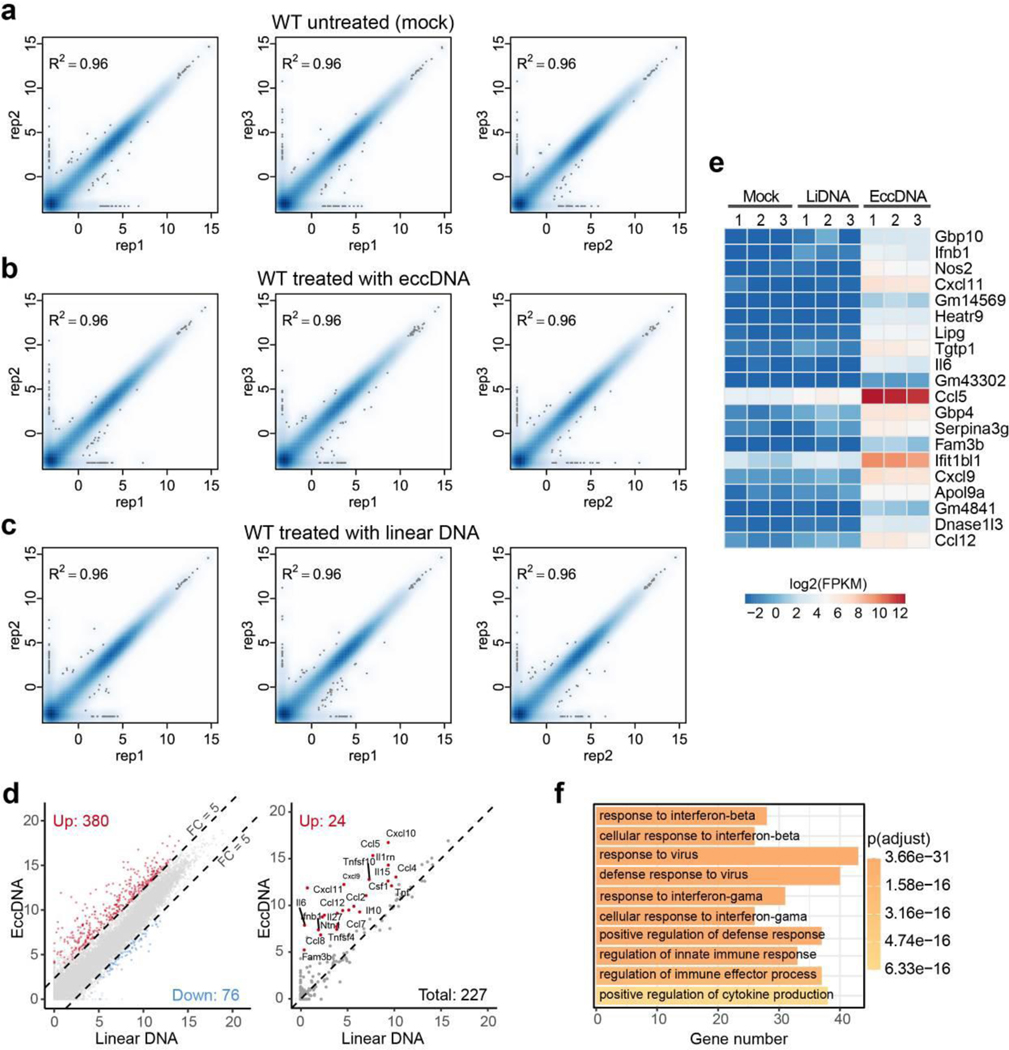
Global transcriptional response to eccDNA in BMDMs **a-c**, Scatter plot of pair-wise comparison of the transcriptomes of three independent BMDM samples of untreated (a), treated with eccDNA (b) or linear DNA (c). The x and y-axis are log2(FPKM+0.1). **d**, Scatter plot showing 380 genes (left panel, red dots) that are significantly induced (FC ≥ 5, adjusted p-value < 0.001; FC and p-value were generated using DESeq2^42^ and p-values were adjusted by IHW^43^) by eccDNA, but not linear DNA, in BMDMs. 24 significantly induced cytokine genes are indicated (right panel, red dots). The x and y-axis are log2-transformed normalized read counts. **e**, Heatmap presentation of the top 20 induced genes. **f**, GO terms enriched in the genes activated by eccDNA treatment in BMDMs. The number of genes in each term and the p-value of the enrichment is indicated.

**Extended Data Fig. 10.**
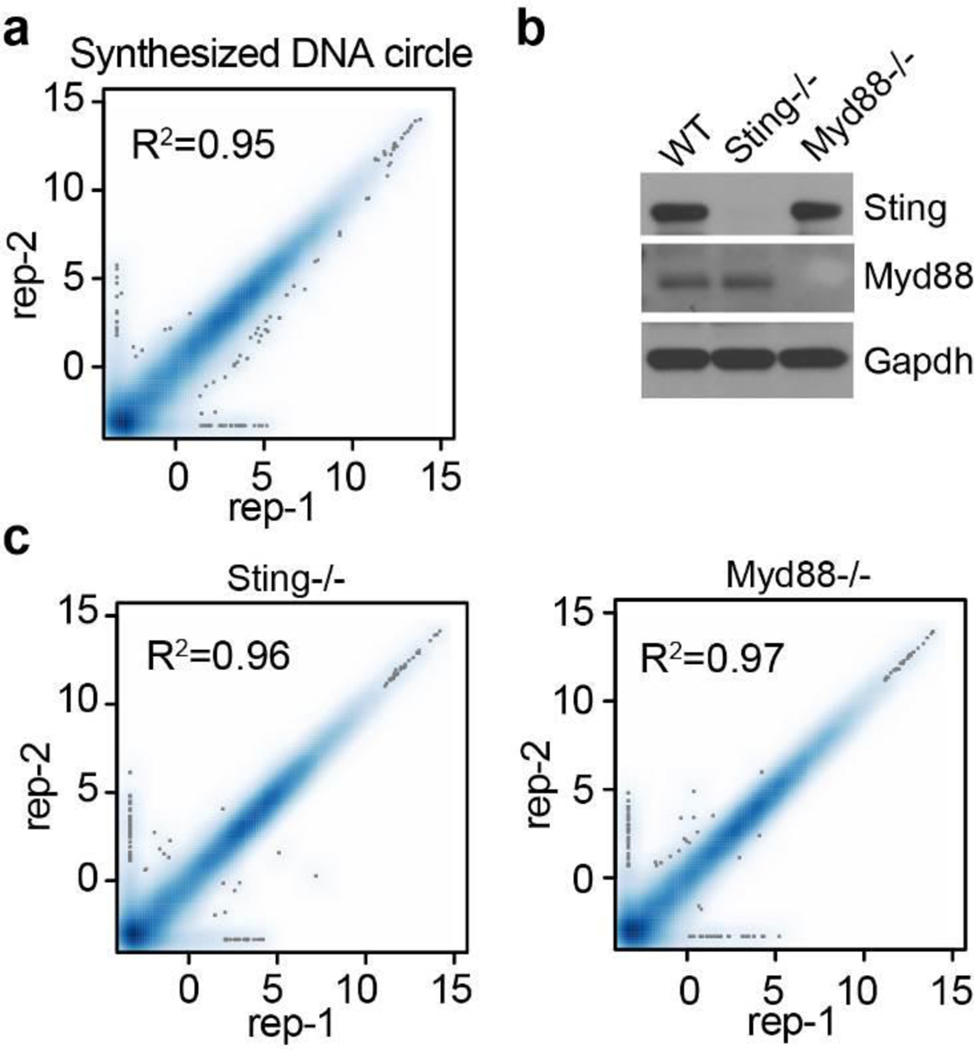
Analysis of the eccDNA sensing pathway **a**, Scatter plot indicates the transcriptomes of the two replicates of synthetic circular DNA treated BMDCs are highly similar. The x and y-axis are log2(FPKM+0.1). **b**, Western blot confirmation of the Sting−/− and Myd88−/− cells. Blots were performed once, but cell genotypes were further confirmed by mRNA sequences from RNA-seq data (data not shown). **c**, Correlation of the transcriptomes of 2 replicates eccDNA treated Sting−/− and Myd88−/− BMDC. The x and y-axis are log2(FPKM+0.1).

**Fig. 5.**
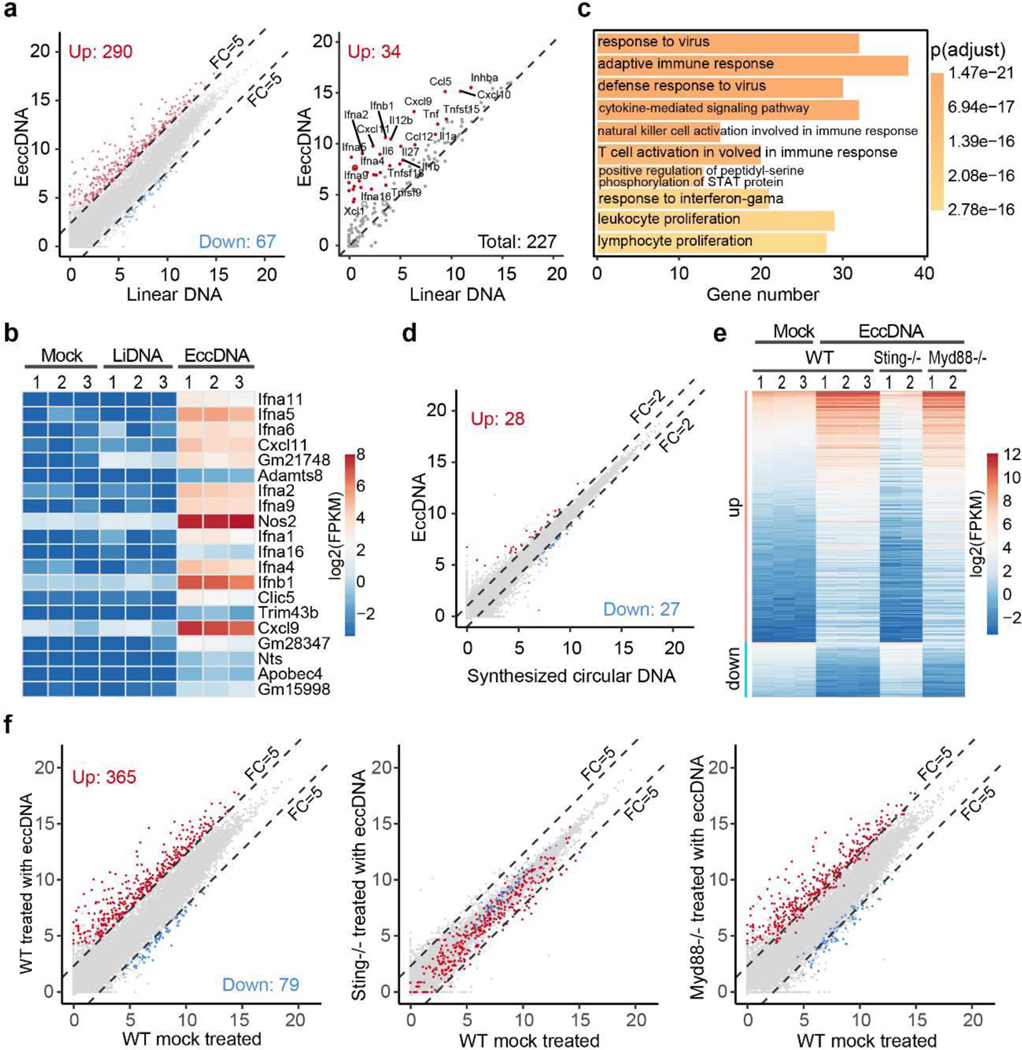
Sting is required for eccDNA induced gene expression **a**, Scatter plot showing 290 genes (left panel, red dots) that are significantly induced by eccDNA, but not linear DNA, in BMDC. 34 significantly induced cytokine genes are indicated (right panel, red dots). FC (fold change). **b**, Heatmap presentation of the top 20 induced genes. **c**, GO terms enriched in the genes activated by eccDNA treatment in BMDC. The number of genes in each term and the p-values of the enrichment are indicated. **d**, Scatter plot indicates that the transcriptomes of BMDC treated with eccDNAs and synthetic circular DNA are highly similar. **e**, Heatmap presentation of the eccDNA responsive genes in control and eccDNA treated BMDC of the indicated genotypes. **f**, Scatter plots comparing the transcriptome affected by eccDNA in BMDC from WT, Sting−/−, and Myd88−/− mice. EccDNA responsive genes in WT BMDC are indicated by red dots (up-regulated, n=365) and blue dots (down-regulated, n=79). The x and y-axis are log2-transformed normalized read counts, p-value were generated using DESeq2^42^ and adjusted by IHW^43^(**a**, **d**, and **f**); FC ≥ 5, adjusted p-value < 0.001 in ( **a** and **f**); FC ≥ 2, adjusted p-value < 0.01 in (**d**).

To assess the global transcriptional effect of eccDNA, we performed RNA-seq analysis of BMDC transfected with purified eccDNAs or sonicated genomic DNA of similar size (Extended Data Fig. 8a**-**c). Comparative analysis indicated that eccDNAs, but not the linear DNA control, significantly increased the expression of 290 genes [FC (fold change) ≥ 5, p-value < 0.001], including 34 cytokines and chemokines (Fig. 5a, Supplemental table 4), under our experimental conditions (30 ng/ml DNA transfected). Importantly, 9 of the top 20 up-regulated genes belong to type I interferons (Fig. 5b). Gene ontology (GO) enrichment analysis revealed that the up-regulated genes are enriched for terms relevant to immune response and related signaling pathways (Fig. 5c), supporting our conclusion that eccDNA is a potent innate immunostimulant that can generally increase innate immune response. Parallel experiments further demonstrated a similar effect of eccDNAs in BMDM (Extended Data Fig. 9a**-**f, Supplemental table 5). Collectively, these data support a higher capacity and potency of eccDNA in triggering a general immune response compared to linear genomic DNA fragments (Fig. 5b). Importantly, this eccDNA property dependents on its circularization, but not its sequence, as transfection of the 200 bp synthetic circular DNA into BMDC triggered a similar transcriptional response when compared to that of purified eccDNAs (Fig. 5d, Extended Data Fig. 10a).

To determine how eccDNA is sensed, two well-known DNA sensing deficient mice, Sting−/− (stimulator of interferon genes)^31^ and Myd88−/− (Myeloid differentiation primary response 88)^32^, were used to generate BMDCs (Extended Data Fig. 10b), which were then subjected to eccDNA transfection. Comparative RNA-seq analysis demonstrated that while loss function of Myd88 did not affect BMDC responds to eccDNAs, loss of Sting function completely abrogated the capacity of BMDC to respond to eccDNAs as almost all the genes normally induced by eccDNA failed to be induced in the absence of Sting (Fig. 5e, f, Extended Data Fig. 10c, Supplemental table 6). These data strongly suggest that the Sting pathway is responsible for sensing eccDNA to mediate its immune response.

Our data indicates that apoptotic oligonucleosomal DNA fragmentation (ODF) is directly linked to eccDNA generation as blocking apoptotic DNA fragmentation abolishes eccDNA production (Fig. 3c, d). This notion challenges the assumption that eccDNAs isolated from a cell population or tissue were equally contributed by each cell^9^. On the contrary, our data suggests that eccDNAs are mostly derived from cells undergo genomic DNA fragmentation. During apoptosis, genomic DNA is first broken into high weight molecular (HWM) fragments (>50 kb) and subsequently undergo ODF to generate oligonucleosomal fragments^18,33^. Similar to ODF in myoblasts^20^ and neuroblastomas under differentiation conditions^34^, we showed that DNase γ is required for ODF and consequent eccDNA generation in mESCs (Fig. 3c, d). This suggests that the eccDNAs that we purified are generated in late stage of apoptosis when ODF occurs^33^, which is consistent with our observation that the great majority (99.5%) of the eccDNAs are within 3 kb in size (Fig. 2b) despite we did identify eccDNAs as long as 10 kb. Although random ligation by *Lig*3 of nucleosomal size DNA fragments in late stage of apoptosis explains the dominant oligonucleosomal sizes and the absence of abundant individual eccDNAs in our study and previous studies^5,9^, it is possible that rare ligation of HWM fragment in early stage of apoptosis might also occur. Our demonstration that *Lig*3 is responsible for nucleosomal size eccDNA generation is consistent with the capacity of *Lig*3 to circularize DNA fragments *in vitro*^35^. Whether *Lig3* is also involved in the biogenesis of large size eccDNAs, the double minutes^36^ and ecDNAs^37^, remains to be determined. Although this study mainly focused on eccDNA generation under apoptotic conditions, we do not rule out the possibility that eccDNAs can also be generated under other conditions that cause genomic DNA fragmentation (eg. replication stress, double strand DNA break, VDJ recombination etc.) and subsequent circularization.

We demonstrated that purified eccDNAs or synthetic circular DNA, but not their linear counterparts, have strong immunostimulatory activity (Fig. 4, 5a-c). Importantly, Sting, but not Myd88, is required to mediate this process (Fig. 5e-f). cGAS-Sting is a well-known intracellular DNA sensing pathway^38^, and DNA sensing of cGAS has been reported to be enhanced by host factors HMGB1 and TFAM, which facilitate DNA bending or form U-shaped structures^39^. Whether these factors are involved in eccDNA-mediated immune stimulation remains to be shown. In addition to the immunostimulatory activity of eccDNA we showed in this study, it is not clear whether eccDNAs from apoptotic cells are link to the oncogene amplification and tumor progression shown for large ecDNAs^37^.

Our demonstration that eccDNAs can dramatically induce type I IFN expression (Fig. 4, 5), combined with previous observations that type I IFNs possess adjuvant activity^40^, and that dying-cells-released DNA mediate aluminum adjuvant activity^24^, prompt us to propose that eccDNAs possess high adjuvant activity. In addition, the existence of eccDNAs in plasma^10^ suggests that eccDNA is highly mobile. Given the increased level of cell free DNA and serum IL-6, TNF-α are good predictors of diseases severity that cause cytokine storm^41^, we suspect that apoptosis-caused eccDNA generation and subsequent induction of cytokine might underlie cytokine storm observed in diseases such as severe sepsis and COVID-19, as these diseases can cause massive cell death. If future studies confirm this notion, the eccDNA biogenesis and sensing pathway revealed in this study could serve as the basis for therapeutic interventions.

In summary, by providing answers to three key questions regarding the origin, biogenesis, and biological function of eccDNAs, our study significantly advances our understanding of eccDNAs. Further characterization of the molecular basis of eccDNA-mediated immune response can provide new insight into innate immunity as well as vaccine design and immunotherapy.

## Materials and Methods

### Cell cultures and apoptosis induction

Mouse ESC-E14 cells were cultured on dishes coated with 0.1% gelatin in standard LIF/serum medium containing mouse LIF (1000U/ml), 15% fetal bovine serum (FBS), 0.1 mM nonessential amino acids, 0.055mM β-mercaptoethanol (BME), 2 mM GlutaMax, 1mM sodium pyruvate and penicillin-streptomycin (PS). HeLa S3 cells were grown in Dulbecco’s modified Eagle’s medium (DMEM) with 10% FBS and 100 U/ml PS. CH12F3 cells were cultured in RPMI1640 with 10% heat inactivated FBS,100 U/ml PS, 2 mM BME, 2 mM GlutaMax. L929 cells were cultured in DMEM supplemented with 10% heat inactivated FBS and 100 U/ml PS.

Apoptotic cell death of mESC were induced with 0.5 μM Etoposide (Selleck), 2 μM Staurosporine (Selleck) for 24 hours, or irradiated without medium using ultraviolet (UV-C) in Stratagene Stratalinker 2400 for 3 mJ and continue culture for 16 hours. CH12F3 apoptotic cell death were treated with 2 μM Staurosporine for 16 hours. Cell viability was analyzed with BD FACSCanto II after staining with Live/Dead Fixable Far Red Dead Cell Stain Kit, FSC-A/SSC-A gates were used to exclude debris and FSC-A/FSC-H were used to gate on singlets, then gate on APC+ as dead cells, data were then analyzed with FlowJo. V10.8.0

### Knockout cell line generation

*DNase γ* and *EndoG* knockout mESC cell lines were generated by CRISPR/Cas9 with transit transfection of px330-mCherry (Addgene 98750). mCherry positive cells were sorted by flow-cell cytometry. Guide RNA targeting sequences and PCR genotyping primers for *DNase γ* and *EndoG* were listed in Supplemental Table 1. Lig1−/− CH12F3 cell line was generated by CRISPR/Gas9 guide RNAs targeting to intron 17 and 19 to delete exon 18–19, which harbors the conserved ligase catalytic site. The deletion resulted in a premature stop codon. NucLig3−/−CH12F3 cell line was generated by specifically deleting sequences containing the nucLig3 start codon and the two subsequent Methionines (Met89-Met144) with CRISPR/Cas9 guide RNAs, while keeping the mtLig3 in frame and functional. Lig4 is a single exon genes, two pairs of guide RNAs were used for two rounds of targeting to obtain homozygous deletion of the entire 2.7 kb exon. Guide RNA sequences were list in Supplemental Table 1. Knockout cell lines were confirmed by immunoblotting.

### EccDNA purification and visualization on agarose gel

To purify eccDNAs, cells were first dehydrated in >90% methanol before crude extrachromosomal DNA were extracted in an alkaline lysis buffer at pH11.8. After neutralization and precipitation, crude extrachromosomal DNA were bound in silica column (QIAGEN Plasmid Plus Midi Kit) in binding buffer (Buffer BB of QIAGEN Plasmid Plus Midi Kit ). Bound DNAs were eluted and digested with *Pac*I (NEB) and Plasmid Safe ATP-dependent DNase (P.S. DNase, Lucigen) for 4–16 hours, and then extracted with Phenol/chloroform/isoamyl alcohol (PCI) solution (25:24:1) in a Phase Lock Gel tube (QuantaBio) to minimize DNA loss. After precipitation with carrier glycogen (Roche) and 1/10 V 3M NaOAc (pH 5.5), the precipitated crude eccDNAs were resuspended in Solution A (One-Step Max Plasmid DNAout, TIANDZ) and the eccDNAs can selectively bound to magnetic silica beads in this solution. The eccDNAs were then eluted with 0.1× Elution buffer (1mM Tris-HCl pH8.0) and the concentration is measured by Qubit dsDNA HS Assay kit (Thermo Fisher).

For comparisons of eccDNA production among treatments or genotypes, both total DNA (Zymo, Quick-DNA microPrep Plus Kit) and eccDNA were purified from equal number of cells, eluted and loaded on agarose gel with equal volume. All DNA, except the PCR genotyping, were resolved with vertical agarose gel electrophoresis and visualized by SYBR Gold (Fisher Scientific, 1:10000) staining.

### Synthetic small DNA circle preparation

Synthetic small DNA circles were prepared by the procedure of Ligase Assisted Minicircle Accumulation (LAMA). Random DNA sequence were generated at (https://faculty.ucr.edu/∼mmaduro/random.htm) with 50% GC content. The isomers of single strand template as well as their amplification primer sets were synthesized from IDT and their sequences are listed in Supplemental Table 3. Products with 5’end phosphate were prepared with 2× Q5 DNA polymerase mixtures (NEB). Equal amount of isomers were added to make 100 μl HiFi Taq DNA ligase reaction mixtures, and placed in thermo cyclers following cycles: 95 °C for 3 min, 60 °C for 10 min and 37 °C for 5 min for at least 10 cycles. Circularized products were recovered by PCR Purification Kit (Qiagen) and digested with Plasmid Safe ATP-dependent DNase (Lucigen) before being recovered with PCR Purification Kit.

### Scanning Atomic force microscope (SAFM) imaging

SAFM imaging of DNA was performed in dry mode^44^. Briefly, one tenth volume of 10× imaging buffer (100 mM NiCl_2_ and 100 mM Tris-HCl, pH8.0) was added to sample to reach final DNA concentration of 0.6–1.0 ng/μl, then 2–5 μl mixture was spread on freshly cleaved mica (Ted Pella) surface. After 2 minutes of incubation, specimen was rinsed twice with 30 μl 2 mM Mg(OAc)_2,_ and specimen was dried before and after rinse with compressed air. Images were taken by using tip C of SNL-10 probe on a Veeco MultiMode AFM with Nanoscope V Controller in “ScanAsyst in Air mode” and processed with Gwyddion 2.50.

### Library preparation and eccDNA sequencing

Nanopore sequencing library for eccDNA was prepared by following Ligation Sequencing Kit (Oxford Nanopore) according to manufactory’s instruction after rolling cycle amplification and debranching. RCA were performed with phi29 DNA polymerase (NEB) with some modification to ensure efficient amplification from 100 pg template per reaction. Briefly, in a 20 μl reaction mixture: 2 μl 10 × phi29 DNA polymerase buffer (NEB), 2 μl 25 mM dNTPs, 1 μl Exo Resistant Random Primer (Thermo Fisher), and eccDNA >=100 pg, were added with ultra-pure H_2_O to 17.6 μl, mixed and incubated at 95 °C for 5 minutes before ramping to 30 °C at 1% Ramp Rate. Then, 1 μl phi29 DNA polymerase, 0.6 μl Pyrophosphatase Inorganic (yeast, NEB), 0.4 μl 0.1M DTT (NEB), 0.4 μl 20 mg/ml BSA (NEB) were added. The reaction mixture was incubated at 30 °C for 10–16 hours. Since high branch structure of RCA products could block nanopore to abolish sequencing, RCA products of eccDNAs were further debranched with T7 Endonuclease I (NEB) before being used for sequencing library construction with Ligation Sequencing Kit (Oxford Nanopore, SQK-LSK109). The library was sequenced in Flow cell (R9.4.1, FL-MIN106D) on MinION according to manufactory’s instruction.

Illumina sequencing library for eccDNA was prepared by Tn5-transposon-based tagmentation with Nextera® XT DNA Sample Preparation Kit according to manufactory’s instruction. Briefly, after validating the purity of eccDNAs with SAFM imaging, 0.5 ng pure eccDNAs were directly tagmented with Tn5 transposase, followed by 12–14 cycles PCR amplification with Illumina sequencing adaptors. Barcoded libraries were pooled and sequenced with Illumina 2500 in 150 PE mode.

### EccDNA sequencing data analyses

#### Nanopore base calling and reads mapping.

The fast5 files generated by Nanopore MinION were fed to Guppy (version 3.5.2) for base calling. The parameters used for Guppy were: --flowcell FLO-MIN106 --kit SQK-LSK109 --qscore_filtering --calib_detect --trim_barcodes --trim_strategy dna --disable_pings --device auto --num_callers 16. The generated reads in fastq format were further processed by porechop (version 0.2.4) to remove adaptor sequences for each read with parameters: --extra_end_trim 0 --discard_middle. To reduce artifacts due to misalignment during reads mapping, we compiled a customized reference mouse genome (mm10combine) based on mm10 reference sequences. Briefly, we downloaded all the nucleotide sequences from the NCBI NT database (Oct. 30th, 2018) and then the R/Bioconductor genbankr (version 1.10.0) was used to distinguish mouse contigs from the contigs of other species. Based on the contig’s description and manual inspection, we removed all the gene-related contigs, only 15,984 were kept. The selected fasta sequences were extracted using the command “blastdbcmd -db nt_db -entry_batch selected_ids.txt -out selected_ids.fa -outfmt %f”. The fasta sequences were mapped to the mm10 genome. Finally, we selected contigs that couldn’t be mapped, contigs that had less than 50% of their sequenced uniquely mapped and contigs that were uniquely mapped but with a sequencing quality <10. At the end, 103 contig sequences were added to the mm10 to build the mm10combine reference genome. Then the cleaned reads were aligned to mm10combine using minimap2^45^ (version 2.17) with parameters: -x map-ont -c --secondary=no -t 16. The alignments for each read were stored in PAF format.

#### Consensus eccDNA generation.

To obtain the consensus boundary and sequence of each eccDNA from the mapped RCA long reads, we developed a tool (https://github.com/YiZhang-lab/eccDNA_RCA_nanopore) that uses the alignments in PAF file as input and outputs eccDNA fragments composition (chromosome, genomic start and end positions of each fragment), successive fragments coverage (number of passes) and consensus sequence derived from each RCA long read. The sub-reads of each RCA long read could be mapped to one genomic location or multiple locations. The sub-reads with mapping quality lower than 30 were discarded. This tool performed bootstrapping of successive sub-reads in each RCA long read to check whether the order of the mapped genomic locations for each sub-read is concordant with their order in the RCA long read. Due to the inaccuracy and gap-prone property of Nanopore reads, we allowed a maximum of 20 bp offset of the mapped genomic positions (start and end position, respectively) between two sub-reads to be considered as mapping to the same location. Reads with discordant sub-reads order, location or strand would be discarded. The exact boundaries of eccDNA fragments were determined by voting from the sub-reads’ start and end positions, respectively. The boundary positions were further refined by threading the sub-reads to ensure no gaps or overlaps between any successive sub-reads. The number of passes for each eccDNA fragment was calculated as the number of concordant sub-reads mapped to that location. Only eccDNA with at least 2 passes was kept for downstream analysis. Each eccDNA sequence was derived from the reference genome sequence where it mapped to, with sequence variants incorporated. The sequence variants were called from sub-reads mapped to the corresponding location, requiring minimum depth of 4 and minimum allele frequency of 0.75.

#### Genomic distribution of eccDNA.

The eccDNA fragments were piled up across the genome. To remove PCR duplicates, eccDNA fragments with the same chromosome, start and end positions were treated as duplicates and only one was retained. The coverage of unique eccDNA fragments at each base of the genome was obtained using bedtools^46^ (version 2.29.2) and stored in bigwig file. The distribution of eccDNA across each chromosome was plotted using karyoploteR^47^ (version 1.14.1) with the bigwig fie as input.

#### Mapping of Illumina sequencing reads.

Raw Illumina sequence reads were first processed by Trimmomatic^48^ (version 0.39) to remove sequencing adaptors and low-quality reads, using parameters: ILLUMINACLIP:adapters/NexteraPE-PE.fa:2:30:10:1:true LEADING:3 TRAILING:3 SLIDINGWINDOW:4:15 MINLEN:75 TOPHRED33. BWA^49^ MEM (version 0.7.17) with default parameters. Then, the reads were mapped to our customized mm10combine reference genome. Duplicated reads were removed by Picard (version 2.23.4). Reads with mapping quality of at least 60 were considered as uniquely mapped and used for downstream analysis. The genomic coverage was calculated using bamCoverage from deeptools^50^ (version 3.5.0) with binSize 1.

### Western blot analysis

Equal numbers of cells were lysed in NuPAGE LDS Sample buffer (Thermo Fisher) and protein extracts were resolved on SDS-PAGE and transferred to PVDF membrane. Antibodies against *Lig1* (Proteintech, 1:1000), *Lig3* (BD Biosciences, 1:1000), *Lig4* (a gift from David Schatz at Yale, 1:1000), Myd88(ProSci, 1:1000), Sting (Proteintech, 1:1000), and Gapdh (Thermo Fisher,1:20000) were used.

### Bone marrow derived dendritic cells and macrophages preparation and stimulation

Male mice, including WT C57/BL6, Sting−/− (Tmem173gt, Stock No: 017537), and Myd88−/− (Myd88tm1.1Defr, Stock No: 009088) were purchased from Jackson lab and were housed on a 12-h light/dark cycle at 23°C with 45–55% humidity. After at least 7 days of habituation, mice between 8–12 weeks old were used to collect bone marrow cells (BMs) for BMDC and BMDM differentiation. BMDC were differentiated in RPMI1640 medium supplemented with 10% heat inactivated FBS (Sigma), 10 mM HEPES, 1 mM sodium pyruvate, 100 U/ml PS, 2 mM GlutaMax and 20 ng/ml mouse GM-CSF (Peprotech). BMDM were differentiated in DMEM supplemented with 10% heat inactivated FBS, 100 U/ml PS, and 20% L929 conditioned medium. Half of the medium was replaced at day 3 and day 6. Identity of BMDCs and BMDMs were confirmed by CD11c^+^ MHC II^+^ and F4/80^+^ CD11b^+^ respectively after exclude debris with FSC-A/SSC-A gates and subsequent FSC-A/FSC-H gates on singlets, data were analyzed with FlowJo_v10.8.0. For cell stimulation, cells at day 7–9 were seeded in 96 wells-plates at 3.5×10^4^ per well. DNA was transfected to cells with FuGENE HD (Promega) in Opti-MEM (Gibico) according to manufactory’s instructions after measuring their concentrations with Qubit dsDNA HS Assay kit (Thermo Fisher). All transfection were done for 12 hours except indicated, media were collected for ELISA, and cells were lysed with Trizol (Thermo Fisher) for RNA isolation.

### Transfection efficiency assay

To determine the transfection efficiency of linear DNA and circular DNA, a set of primers (sequence see Supplemental Table 2) that contained 5 Phosphorothioate bonds at their 5’end was used to prepare ends protected linear DNA by PCR. To balance the effects of Phosphorothioate bonds, the circular form was prepared with the same number of Phosphorothioate bonds as linear one. Then DNA concentration was determined by Qubit dsDNA HS Assay kit (Thermo Fisher), then transfected to BMDCs as described above with FuGENE HD (Promega). After transfection, cells were rinsed with PBS for 3 times, and lysed in 100 μl lysis buffer [50 mM Tris-HCl pH 8.0, 1 mM EDTA, 0.5% Tween-20, 3 units/ml Thermoliable Proteinase K (NEB, Cat# P8111S)], then incubate at 37°C for 2 hour and followed with 15 minutes incubation at 55°C to inactivate Proteinase K. 4 μl cell lysate were used for qPCR with a set of primers targeting to both linear and circular DNA to determine the transfected DNA level.

### Incubation BMDC with supernatant of apoptotic medium

80–100% confluent wildtype and *DNase γ* −/− cells in 10 cm dishes were washed 3 times with PBS and irradiated with 3 mJ of ultraviolet (UV-C) in Stratagene Stratalinker 2400, 10 ml Opti-MEM (Gibico) were added and cultured for another 48 hours. Medium was centrifuged at 650 g for 5 minutes and supernatant was filtrated with 0.45 μm filters. 400 μl supernatant were treated with or without enzymes (*Pac*I, Plasmid Safe ATP-dependent DNase, RNaseA/T1, or Benzonase as indicated) in 500 ul reactions at 37 °C for 2 hours, then dialyzed (MWCO, 10 kDa) with fresh Opti-MEM at 4 °C overnight to deplete ATP that is required for the activity of Plasmid Safe ATP-dependent DNase. Then 100 μl per well of dialyzed supernatant were added to BMDCs in 96 wells plate, equal volume of fresh Opti-MEM were parallelly added to separated wells as mock controls, 12 hours later, cells were collected for RT-qPCR analysis, and presented as relative mRNA level to that of mock controls after normalizing to *Gapdh*.

### RNA isolation, RT-qPCR, RNA-seq and ELISA analyses

Cellular RNA was isolated with Zymo Direct-zol RNA Miniprep kit. Complementary DNA was synthesized with SuperScript III and qPCR was performed with Fast SYBR Green Master Mix (Thermo Fisher). The primer sequences for qPCR of each genes are listed in Supplemental Table 2. Gene induction level were presented as relative fold change (FC) to mock treatment after normalizing to *Gapdh*. Bulk RNA-seq libraries were prepared by following the NEBNext Ultra Directional RNA Library Prep Kit for Illumina (NEB, catalog no. E7420S). For ELISA analysis, ELISA kits of IFN-β, IL-6 and TNF-α were obtained from Biolegend, IFN-α ELISA kits were from PBL Assay Science, assays were performed according to manufactory’s instructions. Appropriate volumes of culture medium were used to ensure that the readouts are within the range of the standard curve.

### RNA-seq data analysis

For RNA-seq data, adaptors and low-quality reads were trimmed using Trimmomatic^48^ (version 0.39) with parameters: ILLUMINACLIP:adapters/TruSeq3-PE.fa:2:30:10:1:true LEADING:3 TRAILING:3 SLIDINGWINDOW:4:15 MINLEN:50 TOPHRED33. The cleaned paired-end reads were aligned to mm10 reference genome with GENCODE^51^ mouse gene set M24, using STAR^52^ (version 2.7.6a) with parameters: --outSAMunmapped Within --outFilterType BySJout --outSAMattributes NH HI AS NM MD --outFilterMultimapNmax 20 --outFilterMismatchNmax 999 --outFilterMismatchNoverReadLmax 0.04 --alignIntronMin 20 --alignIntronMax 1000000 --alignMatesGapMax 1000000 --alignSJoverhangMin 8 --alignSJDBoverhangMin 1 --sjdbScore 1 --outSAMtype BAM SortedByCoordinate --quantMode TranscriptomeSAM. RSEM^53^ (version 1.3.3) was used to quantify gene expression levels using the reads aligned to transcriptome in bam file as input, with parameters: --alignments --estimate-rspd --calc-ci --no-bam-output --seed 12345 --ci-memory 30000 --paired-end --strandedness reverse. The differential expressed genes were identified using DESeq2 package^42^.

### eccDNA linearization

EccDNA linearization was performed by sequential treatment of eccDNAs with the nickase fnCpf1 ^37^ (Applied Biological Materials) and single strand DNA-specific nuclease. 50 ng eccDNAs were nicked in a 20 μl reaction that contained 1/8 volume of 8× fnCpf1 linearization buffer (160 mM HEPES pH7.5, 1.2 M KCl, 4 mM DTT, 0.8 M EDTA, 80 mM MnCl_2_) and 1 μl fnCpf1. After incubating at 37 °C for 1 hour, the treated eccDNAs were extracted with Phenol/chloroform/isoamyl alcohol (PCI) solution (25:24:1) in a Phase Lock Gel tube (QuantaBio) and precipitated at −80 °C with carrier glycogen (Roche) and 1/10 V 3M NaOAc (pH 5.5). Nicked eccDNAs were linearized in 10 μl reaction that include 2 μl 5× buffer (0.25 M NaOAc pH 5.2, 1.4 M NaCl, 25 mM ZnSO_4_), 1 μl S1 Nuclease (Thermo Fisher) at 37 °C for 5 min. The reaction was stopped by adding 40 μl 10 mM Tris-HCl (pH8.0), and the linear eccDNA were immediately recovered by 75 μl SPRIselect beads (Beckman Coulter). Successful linearization of eccDNAs was confirmed by efficient digestion with Plasmid Safe ATP-dependent DNase (Lucigen).

### Statistics

Ordinary One-way ANOVA and Two tailed unpaired *t* tests were performed with GraphPad Prism 9.

## Extended Data

## Supplementary Material

Supplemental Table 1

Supplemental Table 2

Supplemental Table 3

Supplemental Table 4

Supplemental Table 5

Supplemental Table 6

## Data Availability

The eccDNA sequencing data and RNA-seq data have been deposited in the Gene Expression Omnibus (GEO) with accession number GSE165919.
